# Differed Growth Stage Dynamics of Root-Associated Bacterial and Fungal Community Structure Associated with Halophytic Plant *Lycium ruthenicum*

**DOI:** 10.3390/microorganisms10081644

**Published:** 2022-08-15

**Authors:** Yan Li, Xuemin He, Hongfei Yuan, Guanghui Lv

**Affiliations:** 1 College of Ecology and Environment, Xinjiang University, Urumqi 830046, China; 2 Key Laboratory of Oasis Ecology, Ministry of Education, Urumqi 830046, China; 3 Xinjiang Jinghe Observation and Research Station of Temperate Desert Ecosystem, Ministry of Education, Urumqi 830046, China

**Keywords:** saline soil, microbial community structure, growth cycle, root compartments, host effect

## Abstract

*Lycium ruthenicum*, a halophytic shrub, has been used to remediate saline soils in northwest China. However, little is known about its root-associated microbial community and how it may be affected by the plant’s growth cycle. In this study, we investigate the microbial community structure of *L. ruthenicum* by examining three root compartments (rhizosphere, rhizoplane, and endosphere) during four growth stages (vegetative, flowering, fruiting, and senescence). The microbial community diversity and composition were determined by Illumina MiSeq sequencing of the 16S V3–V4 and 18S ITS regions. Proteobacteria, Actinobacteria, Bacteroidetes, Planctomycetes, and Acidobacteria were the dominant bacterial phyla, while Ascomycota, Basidiomycota, and Mortierellomycota were the most dominant fungal phyla. The alpha diversity of the bacterial communities was highest in the rhizosphere and decreased from the rhizosphere to the endosphere compartments; the fungal communities did not show a consistent trend. The rhizosphere, rhizoplane, and endosphere had distinct bacterial community structures among the three root compartments and from the bulk soil. Additionally, PERMANOVA indicated that the effect of rhizocompartments explained a large proportion of the total community variation. Differential and biomarker analysis not only revealed that each compartment had unique biomarkers and was enriched for specific bacteria, but also that the biomarkers changed with the plant growth cycle. Fungi were also affected by the rhizocompartment, but to a much less so than bacteria, with significant differences in the community composition along the root compartments observed only during the vegetative and flowering stages. Instead, the growth stages appear to account for most of the fungal community variation as demonstrated by PCoA and NMDS, and supported by differential and biomarker analysis, which revealed that the fungal community composition in the rhizosphere and endosphere were dynamic in response to the growth stage. Many enriched OTUs or biomarkers that were identified in the root compartments were potentially beneficial to the plant, meanwhile, some harmful OTUs were excluded from the root, implying that the host plant can select for beneficial bacteria and fungi, which can promote plant growth or increase salt tolerance. In conclusion, the root compartment and growth stage were both determinant factors in structuring the microbial communities of *L. ruthenicum*, but the effects were different in bacteria and fungi, suggesting that bacterial and fungal community structures respond differently to these growth factors.

## 1. Introduction

A growing body of research has been highlighting the varied contributions of root-associated microbial communities from vital ecosystem functions to the improvement of aboveground agricultural productivity. Many of these interactions occur in three spatially distinct root compartments that have been defined and studied intensively: the soil adjacent to the root (rhizosphere), the root surface (rhizoplane), and the root interior (endosphere) [1,2].

Plant roots influence the composition of their surrounding microbiomes through a variety of factors. Host plant genetics, age, and growth stage, as well as environmental factors and foreign chemical agents, affect the microbial population [3,4,5,6,7]. This leads to significant diversity with the microbial composition varying throughout the rhizosphere, rhizoplane, and endosphere compartments, as well as in the surrounding bulk soil [3,4]. A detailed characterization of the core root microbiome showed that the dominant phyla within the endosphere are much less diverse than the phyla in the rhizosphere [1,2,3]. These symbiotic microorganisms can have beneficial, neutral, or detrimental impacts on plant growth. The most attractive members colonize in the rhizosphere and endosphere, and make up beneficial microbiomes that enhance plant growth and development. Plants can benefit from direct functions such as nitrogen fixation [8], phytohormone production, and mineral nutrient provision [9]. The benefits can also be indirect; the decomposition of phytotoxic compounds and the inhibition of pathogens create more amenable environments [10], and many microbial metabolites have been suggested to increase resistance or tolerance during drought or hypersaline conditions [11,12].

This population, however, is not only affected by the root compartment but by plant growth as well. Bacterial and fungal communities in the rhizosphere change with the growth cycle in both abundance and structure [4,5,13]. Such transformations may be induced by changes in the root physiology, and the quality and quantity of root exudates, but the mechanisms are still unclear as we do not yet understand the dynamics of these microbial assemblies over the plant life cycle.

Previously, Edwards et al. characterized the temporal progression of the rhizosphere-endosphere microbiota of rice [3]. This study found that the endosphere microbiota reaches a steady-state in two weeks, and a later study revealed the dynamic and successional progression of the microbiota across the three root compartments over the life cycle [4]. However, these studies are not representative of the current state of the field. Most studies that have followed the development of root-associated microbiota throughout the plant life cycle have been limited, mainly characterizing the microbial dynamics of the rhizosphere in regards to glycophytes and crops [4,14,15]. There is little information available on the spatiotemporal dynamics of root-associated microbiota, and even less so for the halophyte root endosphere.

*Lycium ruthenicum* is a halophyte that is often found in saline deserts and sands across Europe, Central Asia, the southern part of Russia, and Northwest China. It is capable of absorbing salt from its crown and the rhizospheric soil, which consequently reduces the salt concentration of the surrounding soils [16]. Therefore, they are used as pioneer plants to improve barren hills and saline lands. The composition and dynamics of the *L. ruthenicum* rhizobial microbiome have not been deeply investigated until now. In this study, we investigate the structure of the root-associated microbial community surrounding roots (the rhizosphere, rhizoplane, and endosphere) over four growth stages (vegetative, flowering, fruiting, and senescence stages) using the Illumina MiSeq sequencing platform. The objectives were: (1) to survey the variation of the microbial community composition variations in three rhizocompartments; (2) to examine the dynamics of the microbial communities during the plant growth cycle; and (3) to examine whether the bacterial and fungal community diversity and structure exhibit similar dynamics.

## 2. Materials and Methods

### 2.1. Study Areas and Sample Collection

The sampling area was located at the Ebinur Lake Wetland Nature Reserve at the Western margin of the Gurbantunggut Desert, Xinjiang, China (44°32′ N–44°36′ N; 83°19′ E–83°33′ E, ~270 m alt.). The Reserve has a typical continental climate; it is dry and windy, with an annual average precipitation of 105 mm, and evaporation of 1315 mm. The soil is highly salinized and alkalized; the average electrical conductivity (EC) and pH value in a 0–10 cm layer are 5.41 ms/cm and 8.77, respectively.

The samples were collected on 28 April, 31 May, 22 July, and 7 October in 2018, corresponding to the vegetative, flowering, fruiting, and senescence stages of *L. ruthenicum* in the study area. Across the four stages, the soil water content (SWC) of the sampling area was 18.6%, 17.3%, 16.3%, and 16.2%, respectively. The average day and five-day (prior to sampling) soil temperatures in a 0–10 cm layer were 15.73 °C and 16.08 °C, 18.65 °C and 18.69 °C, 25.87 °C and 25.72 °C, and 9.57 °C and 13.01 °C.

A total of four healthy individuals with similar heights, ground diameters, and crown widths were selected randomly from the same population at every growth stage for sampling. The roots were excavated; the loosely adhered soil was shaken off and the soil that was attached to the roots (~1 mm) was saved. The roots were cut into short pieces and stored in sterile 50 mL tubes. The bulk soil samples were collected from a 0–30 cm layer of soil approximately 20–50 cm away from the plant. The samples were immediately transported to the laboratory on ice. The bulk soil samples were split into two parts: one half was reserved for chemical property determination, and the other half was transferred into 15 mL tubes and stored at 4 °C until DNA extraction was performed on the same day.

Soils from the rhizosphere (R), rhizoplane (P), and endosphere (E) were separated, and the roots were processed following a protocol that was outlined by Edwars and colleagues [3]. The roots of each sample were vigorously washed with approximately 20–30 mL of sterile phosphate-buffered saline (PBS) solution to remove all the soil from the root surface. The resulting soil solutions were saved in 50 mL tubes and stored as rhizosphere soil samples. The rhizoplane soil compartments were harvested with a sonication protocol to strip the rhizoplane microbes from the root surface. The washed roots were submerged in PBS and sonicated for 60 s at 50–60 Hz. The sonicated roots were collected and immediately transferred into new sterile tubes. The remaining PBS fraction from sonication was kept as the rhizoplane compartment sample. The rhizosphere and rhizoplane soil solutions were filtered through a sterile single-layer mesh to remove any root material. The soil solutions were then centrifuged at 10,000× *g* for 2 min; the resulting pellets were reserved and most of the supernatant was discarded. The endosphere compartment samples were obtained from the same roots after two additional sonication procedures using fresh PBS solution each time as described above. DNA was extracted from the sonicated roots to analyze the endospheric microbiome. Subsamples of the rhizocompartment soil were collected in sterile tubes and stored at −80 °C until analysis.

### 2.2. Soil Chemical Properties Determination

The soil moisture was calculated from the weight difference of the soil that was weighed before and after drying at 105 °C for 48 h. The soil pH was determined using a DDSJ-319L electrode pH meter (INESA Co., Ltd., Shanghai, China) in a 1:2.5 soil/water (*w*/*v*) suspension. The total organic carbon (TOC) was estimated using a UV-1200 Spectrophotometer (Mapada, Shanghai, China), after the soil samples were oxidized with K_2_Cr_2_O_4_. The total nitrogen (TON) was determined using the Kjeldahl method. The concentration of total phosphorus (TP) was determined by Mo-Sb colorimetric analysis with UV-1200 after the soil was digested with a HClO_4_-H_2_SO_4_ solution for 60 min.

### 2.3. DNA Extraction, PCR Amplification, and Sequencing

The rhizosphere soil solutions were concentrated by pipetting 2 mL of the PBS-rhizosphere soil mixture into a 2 mL tube and centrifuging for 60 s at 10,000× *g*. The soil fraction was reserved and the supernatant was discarded. The rhizoplane compartment samples were concentrated in the same manner, except all 15 mL of the sample were concentrated in the same 2 mL tube using multiple centrifugations. The sonicated roots were surface-sterilized with 70% ethanol and homogenized for endosphere DNA extraction. Approximately 0.5 g of fresh bulk soil was used for soil DNA extraction. The genomic DNA for each sample was then extracted using the Omega Soil DNA Kit (Omega Bio-tek Inc., Norcross, GA, USA), and quantified using a Nanodrop 2000 spectrophotometer (Nanodrop Technologies, LLC, Wilmington, DE, USA).

The 16S rDNA V3-V4 region and 18S rDNA ITS region were amplified sequentially in a single reaction containing 15 μL of 2 × Taq Master Mix (Thermo Fisher Scientific, Waltham, MA, USA), 1 μL of each primer (10 μM), and 20 ng of DNA template (30 μL total). First, the 16S V3–V4 region was amplified with barcode-fused primers 341F (gcctacgggnggcwgcag) and 805R (gactachvgggtatctaatcc). The PCR program began with an initial denaturation step at 94 °C for 3 min; followed by 5 cycles of 94 °C for 30 s, 45 °C for 20 s, and 65 °C for 30 s; then 20 cycles of 94 °C for 20 s, 55 °C for 20 s, and 72 °C for 30 s; and a final extension step was held at 72 °C for 10 min. Then, the 18S rDNA ITS region was amplified using Illumina bridge PCR-compatible primers, and the following PCR program: 5 cycles of 95 °C for 30 s, 95 °C for 15 s, 55 °C for 15 s, 72 °C for 30 s, and a final extension for 5 min. The PCR products were visualized using electrophoresis on 1.5% agarose gels, purified using DNA Clean Beads (Vazyme, Nanjing, China), and then quantified using a Nanodrop 2000 spectrophotometer. Finally, 10 ng of DNA from each sequencing sample was sequenced with the Illumina MiSeq platform at the Sangon Technology Co., Ltd. (Shanghai, China), resulting in a total of 64 and 48 samples that were sequenced for 16S and 18S, respectively.

### 2.4. Sequence Analysis

The raw sequence data were first quality controlled as described in [17]. Paired-end reads were merged into sequences with PEAR [18], and the maximum mismatch rate of the overlapping areas was constrained to 0.1. Sequences that were shorter than 200 bp or containing the ambiguous base Ns were removed. Chimeras were identified by UCHIME [19] and discarded. The filtered sequences were then clustered into operational taxonomic units (OTUs) at 97% identity. The taxonomies of representative OTUs were annotated according to their RDP classifier, and BLAST was performed against the Silva and NCBI databases [20]. OTUs with an RDP classification threshold that was below 0.8, or with <90% identity and coverage were denoted as unclassified. All OTUs that were identified as plastid and mitochondrial DNA were removed.

### 2.5. Statistical Analysis

Low abundance OTUs (total counts < 5 across all samples) were eliminated and the remaining OTUs were used for downstream analyses. Mothur 1.30.1 [21] was used for rarefaction analysis, and the vegan 2.4.2 package was used to calculate the alpha diversity indices and Good’s coverage. A one-way analysis of variance (ANOVA) was used to determine the differences and their significance among the three compartments and four growth stages. Histograms were generated using R to illustrate the community structure. Principal coordinates analysis (PCoA) was carried out to evaluate the community beta diversity, and weighted UniFrac (WUF) distances were used to visualize the separation between the communities from the compartments and growth stages. Non-metric multidimensional scaling (NMDS) ordinations were also constructed for further beta-diversity analysis.

An analysis of similarities (ANOSIM) and permutational multivariate analysis of variance (PERMANOVA) was carried out to evaluate the similarities in the community structure, as well as the effects of different growth cycles and spatial compartments on community composition. Differential abundance analysis was conducted to identify OTUs that were correlated with community separation between the compartments or growth stages by fitting a generalized linear model with a negative binomial distribution to the normalized values for each of the OTUs; OTU counts from the bulk soil or vegetative stages were used as controls. DESeq2 [22] was used to calculate the differential abundance (a log10-fold change in the relative abundance of each OTU) between the compartments or growth stages. We defined OTUs with an absolute value of log10-fold change >2 and *p* < 0.05 as enriched or depleted OTUs. FDR-adjusted *p* values were estimated to control for false-positive OTUs. Although we found that the adjusted *p* value = 1 in a large part of the samples, we still used this *p* value as it may be due to the small number of unpooled biological replicates (4 replicates per rhizocompartment in each growth stage). The overlap of those OTUs were defined by a Venn diagram. To further explain the differences between the compartments and growth stages, compartment-specific and growth stage-specific biomarkers were identified by using the linear discriminant analysis (LDA) effect size (LefSe) with LDA > 3 [23].

## 3. Results

### 3.1. Soil Chemical Properties

The pH, EC, and TP of the bulk soil gradually increased from the vegetative to the senescence stage, whereas the SWC decreased in this period. The TOC and TN initially decreased from the vegetative to the flowering stage, then later increased with TOC peaking in the fruiting stage, and the TN peaking in the senescence stage. In contrast to the bulk soil, the rhizosphere had a lower pH, significantly higher TOC and TN, and insignificant differences in TP (Table S1).

### 3.2. Statistic of Sequencing Data

Following the removal of chimera and organelle sequences, and other quality control measures, 613 to 78,902 high-quality 16S sequences and 15,650 to 81,526 high-quality 18S sequences were obtained per sample, with averages of 39,770 and 57,770 sequences, respectively. A range of 159 to 2946 16S OTUs per sample with an average of 1533, and a range of 50 to 1404 18S OTUs per sample with an average of 523 were identified based on the uniformed 20,000 sequences per sample at a cut-off of 97% sequence similarity. Good’s coverage (all > 95.81%) (Tables S2 and S3) and rarefaction curves (Figures S1 and S2) suggested that the sequences for each sample were sufficient to characterize the microbial communities in the studied soils. After further exclusion of low abundance OTUs, a total of 12,183 16S OTUs and 5765 18S OTUs remained for subsequent analysis. The relative abundance and taxonomic classifications of all OTUs are listed in Tables S4 and S5.

**Figure 1:**
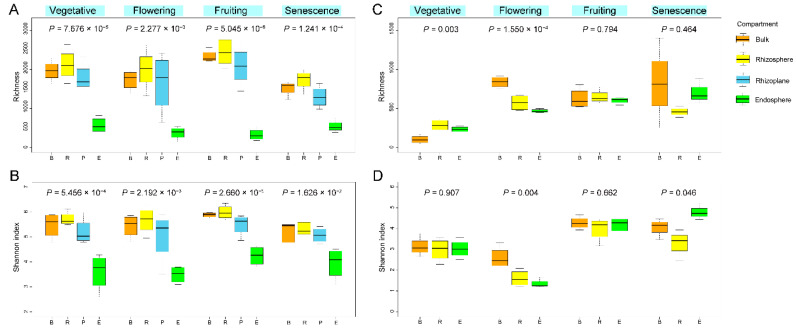
Estimated OTUs richness and Shannon diversity of rhizospheric compartments in each growth stage for bacterial (**A**,**B**) and fungal (**C**,**D**) communities, indicating a general decreasing gradient in the microbial diversity from the rhizosphere to the endosphere. The *p* values indicate significance between the four compartments.

The richness and diversity of bacterial communities in the three compartments had significant differences throughout the whole growth cycle. The OTU richness and diversity of bacteria appeared to decrease with increased proximity to the root, with the highest diversity found in the rhizosphere and the lowest in the endosphere (Figure 1A,B). Fungal communities, however, did not show such consistent patterns. The differences in the community composition among the three compartments were significant during the vegetative and flowering stages, but not in the fruiting and senescence stages. Likewise, fungal richness and diversity were higher in the rhizosphere than in the endosphere during the vegetative, flowering, and fruiting stages, but the opposite was true in the senescence stage (Figure 1C,D).

### 3.3. Microbial Community Structure of Bacteria and Fungi

#### 3.3.1. Bacterial Community Structure

**Figure 2:**
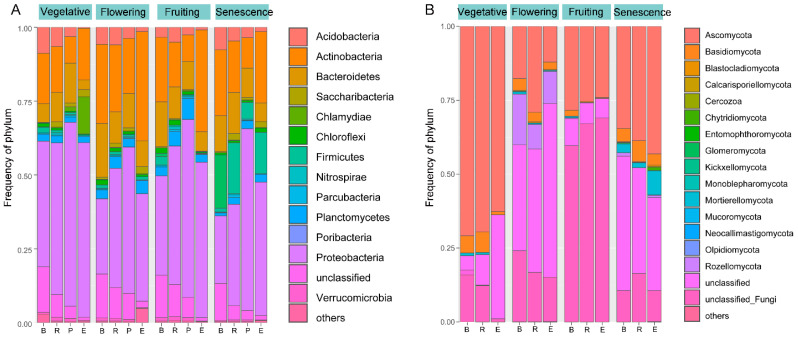
Histograms of the phyla abundances in bacterial community (**A**) and fungal community (**B**). R, rhizosphere; P, rhizoplane; E, endosphere; B, bulk soil; unclassified, has no assignment of affiliation at the relevant taxonomic level.

In total, 40 bacterial and archaeal phyla (Figure 2A, Table S6) and 15 fungal phyla (Figure 2A, Table S7) were identified. Among the 40 prokaryotic phyla, Proteobacteria, Actinobacteria, Bacteroidetes, Planctomycetes, and Acidobacteria were dominant in nearly all of the samples, accounting for 64.52% to 98.26% of all the sequences in each sample. Proteobacteria was the most abundant phylum; its relative abundance increased from the bulk soil to the rhizosphere, then decreased slightly in the rhizosphere and rhizoplane. Actinobacteria was the most abundant in the endosphere (17.51% to 36.97%), and its abundance decreased from the bulk soil to the rhizosphere and rhizoplane. The abundance of Bacteroidetes was much lower in the endosphere (3.31%~8.74%) in comparison to the other two compartments and the bulk soil. The abundance of Acidobacteria gradually decreased from the bulk soil to the endosphere (Table S6). Firmicutes showed a trend that was similar to Acidobacteria in the first three growth stages, but its relative abundance increased nearly 3.5 to 13.8 times of the bulk soil in the three root compartments during the senescence stage. At the genus level, *Pelagibius* was the most abundant, with an average of 3.81% across all the samples, and significantly enriched in rhizosphere, rhizoplane, and endosphere. *Haliea*, *Gp10*, *Gracilimonas*, *Geminicoccus*, *Thioprofundum*, *Porphyrobacter*, *Actinophytocola*, *Arenicella*, *Nocardioides*, and *Pseudomonas* were also abundant genera with average abundance that was higher than 1%.

Our analyses suggest that the composition of the microbiome is affected by the distance to the plant root, and some genera exhibit general preferences. For example, some genera (e.g., *Gp10*, *Gp21*, *Salisaeta*, *Nitriliruptor*, *Rhodoligotrophos*, *Phycisphaera*, *Deferrisoma*, *Aciditerrimonas*, *Euzebya*, *Alterococcus*, *Gillisia*, and *Gracilimonas*) decreased in abundance from the bulk soils and rhizospheric soils as it approached the endosphere regardless of the growth cycle. In contrast, others (e.g., *Porphyrobacter*, *Arenicella*, *Nocardioides*, *Mycobacterium*, *Nocardia*, and *Actinophytocola*) increased in abundance with increased proximity to the endosphere. *Nocardioides*, *Methyloceanibacter*, and *Actinophytocola* were significantly enriched in the endosphere, whereas *Halomonas*, *Nitrospira*, and *Geminicoccus* were significantly depleted. *Bacillus* was enriched in the rhizosphere and rhizoplane, but in low abundance in the endosphere. We also observed patterns that correlated with the growth stage, as *Mesorhizobium* was significantly enriched in the senescence stage (Table S6).

#### 3.3.2. Fungal Community Structure

Ascomycota, Basidiomycota, and Mortierellomycota were the dominant fungal phyla in most of the samples, accounting for 14.93% to 77.59% of all sequences. A large proportion of the sequences (22.32% to 75.59%) were assigned as unclassified fungi, especially in the flowering to senescence stages (Figure 2B, Table S7). At the genus level, *Zopfiella*, *Aporospora*, *Fusarium*, *Corollospora*, *Aspergillus*, *Cephalotrichum*, *Halosarpheia*, and *Mortierella* were the most abundant groups (>1% of the total sequences) (Table S7). Unlike bacteria, the fungal phyla and genera did not exhibit a consistent pattern across the three root compartments, but high variation was observed in rhizocompartments throughout the growth season. For example, Basidiomycota decreased from the rhizosphere to the endosphere through the progression of the growth season, whereas Ascomycota and Mortierellomycota decreased from the rhizosphere to the endosphere in the vegetative, flowering, and fruiting stages, but the opposite in the senescence stage (Figure 2B, Table S7).

### 3.4. Microbial Community Structure Exhibiting Distinct and Overlapping in Three Spatial Root Compartments

**Figure 3:**
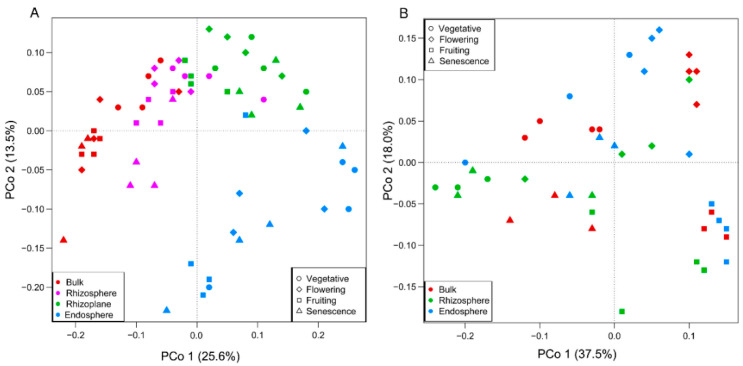
PCoA using the WUF metric indicates that the largest separation between the microbial communities is spatial proximity to the root compartment for bacterial communities (**A**) and growth stage for fungal communities (**B**). The circle, square, triangle, and diamond symbol represents samples collected from different growth stage; red, pink, green and blue colored symbols represent different rhizocompartment. Color and symbol combination represents rhizocompatrment samples in each growth stage.

**Figure 4:**
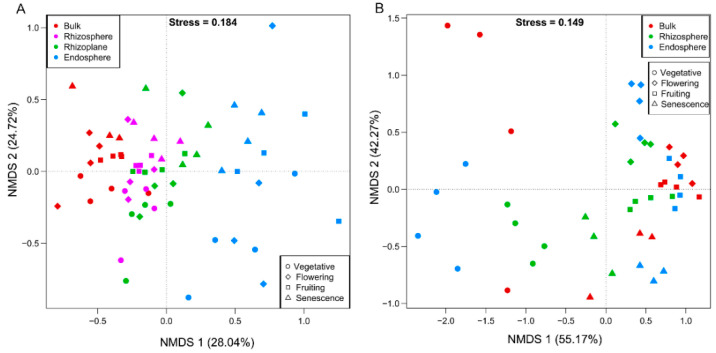
Non-metric multi-dimensional scaling plots indicate that the largest separation between microbial communities is spatial proximity to the root compartment for bacterial communities (**A**) and growth stage for fungal communities (**B**). The meanings of symbols and color are the same with Figure 3.

Both ANOSIM and PERMANOVA revealed differences in the microbial communities across the different spatial components at the same growth stage or at different growth periods in the same rhizocompartment (Table 1). The bacterial communities structure showed a significant difference between the three root compartments across the four growth stages. The patterns that bacterial communities differed significantly between root-associated compartments in the four growth stages was further supported by the PCoA that was based on the weighted UniFrac (WUF) metric and NMDS (Figure 3A and Figure 4A). Whereas for fungi, significant rhizocompartmental differences were observed in the vegetative and flowering stages, but not in the senescence stage. Moreover, the significant difference in the fruiting stage was only supported by PERMANOVA. The PCoA and NMDS also did not reveal any apparent patterns of fungal communities by root compartment, but there were some that were identified when analyzing by growth stage (Figure 3B and Figure 4B).

#### 3.4.1. Association of Significantly Enriched Bacterial OTUs with Different Rhizocompartments

**Figure 5:**
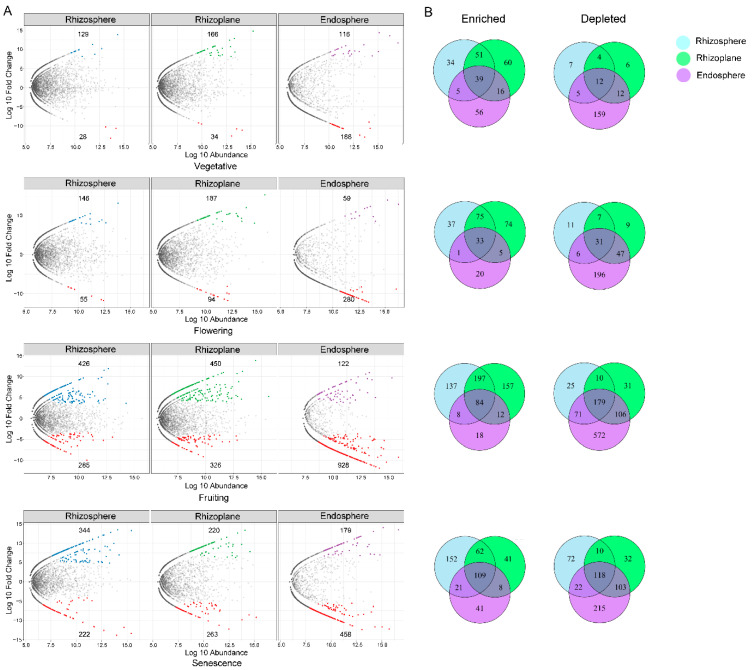
Comparison between the four compartments in each growth stage of bacterial communities. (**A**) Enrichment and depletion of 16S OTUs for each rhizocompartment (Rhizosphere, Rhizoplane, and Endosphere) compared to the bulk soil in the four growth stages as determined by differential abundance analysis. Each point represents an individual OTU, of which the blue, green, and purple points represent enriched OTUs in each compartment compared to the bulk soil, while the red ones indicate depleted OTUs, and the grey points represent no difference in abundance between each root compartment and bulk soil. The position along the y-axis represents the abundance fold change compared with the bulk soil. (**B**) Venn diagrams showing the numbers of differentially enriched and depleted OTUs between each compartment compared with bulk soil.

Differential OTU abundance analysis was conducted to identify OTUs that correlated with community separation between rhizocompartments, using the bulk soil as a control. The rhizosphere was the most similar to the bulk soil, and an enrichment effect of the rhizosphere was implied by the high ratio of statistically significant enriched OTUs in comparison to depleted OTUs (129 vs. 28 in the vegetative stage, 146 vs. 55 in the flowering stage, 426 vs. 285 in the fruiting stage, and 344 vs. 222 in the senescence stage). In comparison, the rhizoplane was enriched for many OTUs while simultaneously had a larger proportion of depleted OTUs (160 vs. 34 in the vegetative stage, 187 vs. 94 in the flowering stage, 450 vs. 326 in the fruiting stage, and 220 vs. 263 in senescence the stage). The endosphere was the most exclusive compartment, enriching 116, 59, 122, 179 OTUs while depleting 188, 280, 928, 458 OTUs in each growth stage, respectively (Figure 5A).

There were overlaps in differentially abundant OTUs between the compartments (Figure 5B). For instance, the OTUs that are enriched in the rhizosphere are not only very successful at colonizing the root, but also a large proportion of these OTUs were enriched in the rhizoplane, endosphere, and/or both (95 out of the 129 OTUs in the vegetative stage, 109 out of the 146 OTUs in the flowering stage, 289 out of the 426 OTUs in the fruiting stage, and 192 out of the 344 OTUs in the senescence stage). Additionally, 39, 33, 84, and 109 OTUs were enriched in all three compartments compared to the bulk soil in the vegetative, flowering, fruiting, and senescence stages, respectively. These enriched OTUs belonged mainly to 69 genera in Actinobacteria, Bacteroidetes, Planctomycetes, Alpha-, and Gamma-Proteobacteria (Tables S8–S11, Figures S3–S6). Of this collection, 24 genera appeared in at least two stages; three genera (*Devosia*, *Pirellula*, *Gimesia*) appeared in three stages, and seven genera (*Actinophytocola*, *Mycobacterium*, *Nocardia*, *Nwocardioides*, *Arenicella*, *Haliea*, and *Porphyrobacter*) were observed in four stages.

The exclusionary effects of each compartment relative to the bulk soil showed that the rhizosphere had a small influence on excluding microbes, as only 28, 55, 285, and 222 OTUs were significantly depleted compared with the bulk soil in the vegetative, flowering, fruiting, and senescence stages, respectively (Figure 5A, Tables S12–S15). Many more OTUs were depleted in the rhizoplane (34, 94, 326, and 263 OTUs in each growth stage, respectively), and even more were reduced in the endosphere (188, 280, 928, and 458 OTUs in each growth stage, respectively). These depleted OTUs were mainly Proteobacteria, Acidobacteria, Actinobacteria, Bacteroidetes, Chloroflexi, Firmicutes, Planctomycetes, and Verrucomicrobia; Euryarchaeota was reduced significantly only in the senescence stage (Tables S12–S15).

There were considerable overlaps in the excluded OTUs from each compartment as well (Figure 5B). A large proportion of the OTUs that were depleted from the rhizosphere were also depleted in the rhizoplane and endosphere communities. The rhizoplane shared 24 of the 188 OTUs that were significantly depleted from the endosphere in the vegetative stage, and in the other three growth stages, 78 out of the 188 OTUs, 285 out of the 925 OTUs, and 221 out of the 458 OTUs, were depleted in both the rhizoplane and endosphere, respectively (Tables S12–S15).

#### 3.4.2. Association of Significantly Enriched Fungal OTUs with Different Rhizocompartments

**Figure 6:**
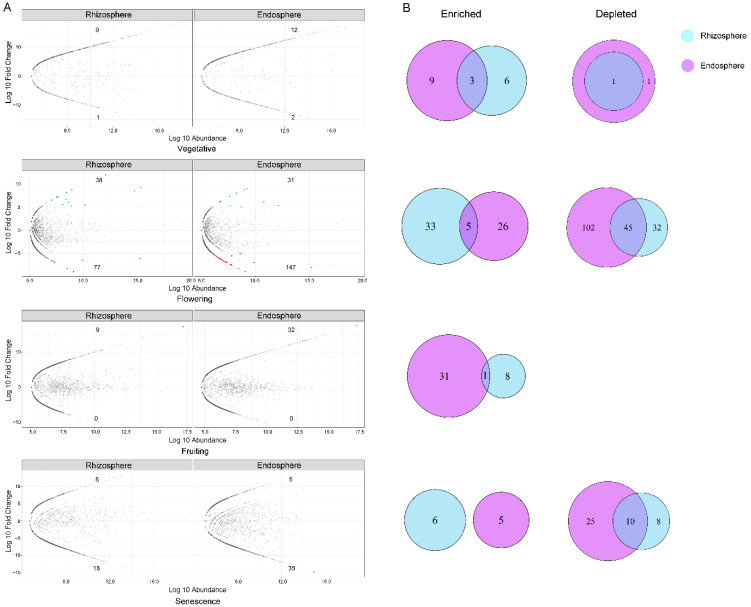
Comparison between two rhizocompartments in each growth stage of fungal communities. (**A**) Enrichment and depletion of 18S OTUs for each rhizocompartment (rhizosphere and endosphere) compared with the bulk soil in four growth stage as determined by differential abundance analysis. Each point represents an individual OTU, of which the blue and purple points represent enriched OTUs in each compartment compared to the bulk soil, while the red ones indicate depleted OTUs, and the grey points represent no difference in abundance between each root compartment and bulk soil. The position along the y-axis represents the abundance fold change compared with bulk soil. (**B**) Venn diagrams showing the numbers of differentially enriched and depleted OTUs between each compartment compared with the bulk soil.

Unlike our observations of bacterial communities, there was only a small number of fungal OTUs that were enriched or depleted in the rhizosphere or endosphere compared to the bulk soil community (Figure 6A), which is consistent with the ANOSIM and PERMANOVA analyses (Table 1), indicating there is a relatively high similarity between the root-associated fungal communities with the bulk soil.

The enrichment effect of the root compartments was observed in the vegetative and fruiting stages, but there were stronger exclusionary effects in the flowering stage and senescence stages. In the vegetative stage, the enriched and depleted OTUs were 9 vs. 1 in the rhizosphere, while it was 12 vs. 2 in the endosphere. The rhizosphere was enriched with *Aporospora*, *Cephalotrichum*, *Chaetothyriales*, *Podospora*, *Halosarpheia*, and *Penicillium*, while the endosphere was enriched with *Aspergillus*, *Aporospora*, *Roussoella*, *Halosarpheia*, and *Phomopsis* (Table S16). In the flowering stage, there were 38 enriched vs. 77 depleted OTUs in the rhizosphere, and 31 enriched vs. 147 depleted OTUs in the endosphere. The OTUs that were enriched in the rhizosphere were affiliated with 13 species, such as *Alternaria chlamydospora*, *Ascobolus crenulatus*, and *Zopfiella marina*; the endosphere was enriched with *Corollospora maritima*, *Halosarpheia japonica*, *Sarocladium subulatum*, *Savoryella appendiculata*, and *Zopfiella marina* (Table S17). In the fruiting stage, 9 and 32 OTUs were enriched in the rhizosphere and endosphere, respectively, with no depleted OTUs. The most abundant genera in the rhizosphere were *Cephalotrichum*, *Fusarium*, *Halosarpheia*, *Penicillium*, and *Sarocladium* (Table S18). There were no shared OTUs between the two root compartments in the senescence stage; the rhizosphere had 6 enriched vs. 18 depleted OTUs, and the endosphere had 5 enriched vs. 35 depleted OTUs (Figure 6A, Table S19).

The overlaps of enriched or depleted OTUs from each compartment were dependent on the growth stage. There were three OTUs belonging to the genera *Aporospora* and *Halosarpheia* that were enriched in both the rhizosphere and endosphere in the vegetative stage. The five OTUs that were enriched in both the rhizosphere and endosphere in the flowering stage were *Corollospora* (*C. maritima*), *Sarocladium* (*S. subulatum*), *Halosarpheia* (*H. japonica*), and *Zopfiella* (*Z. marina*). The shared enriched OTUs in the fruiting stage were marked as unclassified, and there are no shared OTUs between the two compartments in the senescence stage (Tables S16–S19).

The endosphere exhibited a higher exclusionary effect than the rhizosphere. The endosphere had 147 depleted OTUs in the flowering stage and 35 in the senescence stage, whereas the rhizosphere had 77 reduced OTUs in the flowering stage and 18 OTUs in the senescence stage. (Figure 6A). More than half of the depleted OTUs in the rhizosphere were also reduced in the endosphere (Figure 6B). The depleted OTUs in the rhizosphere that coincided with the endosphere were 1 out of 1, 45 out of 77, and 10 out of 18, in the vegetative, flowering, and senescence stages, respectively (Figure 6B). These shared OTUs belonged mainly to the genera of *Metarhizium* (*M. anisopliae*), *Fusarium* (*F. oxysporum*), *Cryptococcus*, *Simplicillium* (*S. lanosoniveum*), *Dekkera* (*D. custersiana*), *Sarocladium* (*S. zeae*), *Geminibasidium*, and *Taifanglania* (Tables S20–S22).

### 3.5. Compartment-Specific Biomarkers Identification in Each Root Compartment

In total, 92, 71, 86, and 107 bacterial clades (phylum to genus) were identified in each growth stage. The total number of bacterial biomarkers that were found in the rhizosphere and rhizoplane were lower in the first three growth stages than in the senescence stage (33, 25, and 19 clades, respectively, vs. 52 clades) (Figures S7–S10).

The discriminating taxonomy groups varied in each compartment. In the vegetative stage, Verrucomicrobiam, Bacteroidetes, and Proteobacteria were the major phyla that contributed to the distinctiveness of the bacterial communities in the rhizosphere, rhizoplane, and endosphere, respectively. Additionally, there were several predominant genera that determined the community dissimilarities in the rhizoplane (e.g., *Geminicoccus* and *Gp10*), rhizosphere (e.g., *Cytophagia*, *Flavobacteriia*, *Pelagibius*, and *Haliea*) and endosphere (e.g., *Nocardiopsis*, *Microbulbifer*, and *Porphyrobacter*) in the vegetative stage (Figure S7). The number and composition of biomarkers changed throughout the flowering to senescence stages, but there were some shared dominant biomarkers in the flowering and fruiting stages such as *Gp10* in the rhizosphere, and *Haliea* and *Pelagibius* in the rhizoplane. Endospheric biomarkers were different in the flowering and fruiting stages; genera *Pseudomonas*, *Ilumatobacter*, *Nocardioides*, and *Glycomyces* were unique to the former stage (Figure S8), and *Lactobacillus*, *Novosphingobium*, *Acinetobacter*, *Sinomicrobium*, *Devosia*, *Methyloceanibacter*, *Porphyrobacter*, *Arenicella*, *Nocardioides*, *Acholeplasma*, and *Actinophytocola* in the latter (Figure S9). Even more unique biomarkers were found in the senescence stage, such as those for genera *Nafulsella*, *Geminicoccus*, *Deferrisoma*, *Pontibacter*, *Bacillus*, and *Planomicrobium* in the rhizosphere; *Devosia*, *Thioprofundum*, *Brevundimonas*, *Halomonas*, and *Mesorhizobium* in the rhizoplane; and *Streptomyces*, *Nocardia*, *Arenicella*, *Nocardioides*, *Porphyrobacter*, *Methyloceanibacter*, and *Haliea* in the endosphere (Figure S10).

The most fungal biomarkers were detected in vegetative stage, while the least in senescence stage. There were 47, 34, 25, and 10 fungal clades that were detected in the vegetative, flowering, fruiting, and senescent stages, respectively. There were fewer biomarkers in the rhizosphere and endosphere compartments (15 and 8 in the vegetative and flowering stages, respectively) than the bulk soil (22 and 26, respectively) during the first two growth stages, but the opposite trend was observed in the last two stages (Figures S11–S14). The phylum Basidiomycota and the species *Vishniacozyma carnescens*, *Filobasidium* sp., and *Aureobasidium namibiae* were discriminating groups in the rhizosphere, while *Aspergillus*, *Halosarpheia*, *Roussoella,* and *Phomopsis* were discriminating biomarkers in the endosphere. In the flowering stage, many biomarkers were identified in the bulk soil, but there much less in the root compartments. *Zopfiella* (*Z. marina*) was identified as in the rhizopshere, while the endosphere was mainly comprised of unclassified fungi. In the fruiting stage, no biomarkers were detected in the endosphere, but genera *Cephalotrichum*, *Halosarpheia*, and *Scedosporium* were significantly abundant in the rhizosphere. The fewest number of biomarkers were in the senescence stage, which included those for *Alternaria*, *Chaetomium*, *Penicillium*, and *Mortierella* in the endosphere.

### 3.6. Growth Stage Dynamics of Microbial Communities in Each Rhizocompartment

#### 3.6.1. Diversity Dynamics along with Growth Stage

**Figure 7:**
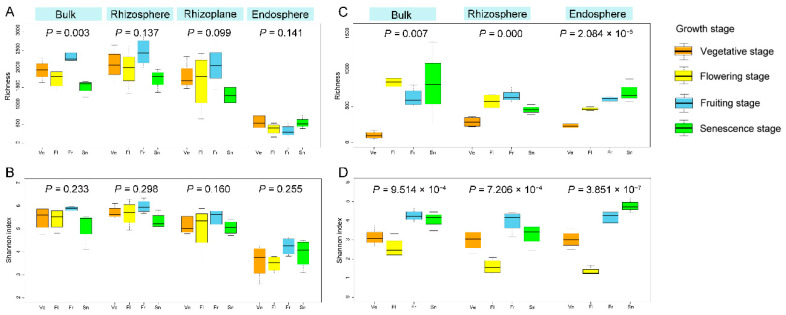
Growth stage changes of the estimated OTUs richness and Shannon diversity for bacterial (**A**,**B**) and fungal (**C**,**D**) communities in the bulk soil, rhizosphere, rhizoplane, and endosphere compartments. The *p* values indicate significance between the four growth stages.

The community diversity responded dynamically to the plant growth stages, but the patterns of change differed between bacteria and fungi, also varied in rhizocompartments. There was no significant difference in the richness and diversity of bacterial communities between the four growth stages in each root compartment (*p* > 0.05), but there were significant differences in the fungal communities in all three compartments (*p* < 0.05). Although it was not significant, the bacterial community diversity (Shannon index) showed an increase from the vegetative stage to the fruiting stage, then decreased in the senescence stage in the four compartments. In fungal communities, the Shannon diversity was higher in the fruiting and senescence stage than in the earlier stages (Figure 7).

#### 3.6.2. Growth Stage Dynamics of Bacterial Community Structure

The growth-stage dynamic was further supported by community composition. ANOSIM and PERMANOVA demonstrated significant growth stage differences in the bacterial community structure in the rhizosphere and endosphere (Table 1); the bacteria dissimilarities among growth stage (PERMANOVA R^2^ = 0.268 in the bulk soil, 0.554 in the rhizosphere, 0.274 in the rhizoplane, and 0.339 in the endosphere) were lower than what was found between the rhizocompartments (PERMANOVA R^2^ = 0.526, 0.527 0.398, and 0.637 in the vegetative, flowering, fruiting, and senescence stages, respectively) (Table 1). These analyses in conjunction with the spatial separation patterns (Figure 3 and Figure 4) suggest that the bacterial communities differed significantly by root-associated compartments, more so than by growth stage, although the growth stage also influenced the bacterial communities.

The dominant phyla during the four plant development stages were similar in each compartment, but there were variances in the relative abundance (Tables S5 and S6). The bulk soil and rhizosphere had Proteobacteria abundance that decreased from the vegetative to senescence stages, but the rhizoplane and endosphere experienced a decreased abundance from the vegetative to fruiting stages, then an increase in the senescence stage. Actinobacteria had the highest abundance in the fruiting stage than the other three stages in all of the rhizocompartments, while Firmicutes were significantly enriched in the senescence stage and its abundance was higher than in the other growth stages in the three root-associated compartments. The three dominant fungal phyla also varied in abundance relative to the growth stage. The abundance of Basidiomycota was reduced significantly from the vegetative to the flowering stage but was then followed by a gradual increase from the flowering stage to the senescence stage.

**Figure 8:**
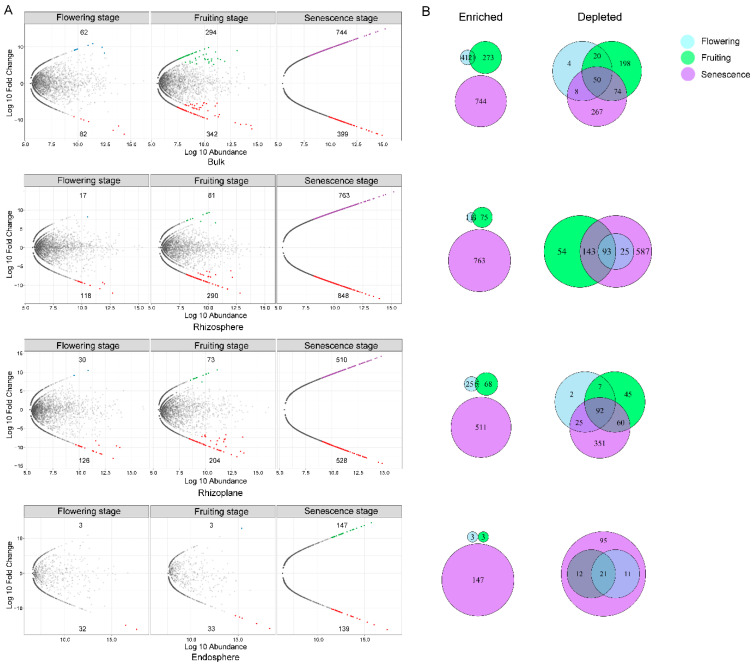
Bacterial community comparison between the four growth stages in each root-associated compartment. (**A**) MA diagrams showing the enrichment and depletion of 16S OTUs for each growth stage (flowering stage, fruiting stage, and senescence stages) compared with the vegetative stage in three rhizocompartments. Each point represents an individual OTU, of which the blue, green, and purple points represent enriched OTUs in the flowering stage, fruiting stage, and senescence stages compared to the vegetative stage, while the red ones indicate the depleted OTUs, and the grey points represent no difference in abundance. The position along the y-axis represents the abundance fold change compared with the bulk soil. (**B**) Venn diagrams illustrating the numbers of differentially enriched and depleted OTUs between each stage compared with the vegetative stage.

Differential analysis revealed that the flowering, fruiting, and senescence stages had a gradual increase of enriched or depleted bacterial OTUs in all the four compartments, but the vegetative stage did not exhibit such a pattern (Figure 8A). This suggests that as the growth cycle proceeded, there were enhanced filter effects from the soil to the root, especially in the senescence stage. For instance, there were 763 enriched and 848 depleted OTUs in the rhizosphere, 510 enriched and 528 depleted OTUs in the rhizoplane, and 147 enriched and 139 depleted OTUs in the endosphere (Figure 8A). The degree of enrichment or depletion decreases with increased proximity to the root, which suggests that plant senescence can have a great effect on the root-associated bacterial community. Compared to the rhizosphere and rhizoplane, the bacterial community in the endosphere remained relative stable from the vegetative to fruiting stages as only fewer OTUs were significantly enriched or reduced.

There is only a small degree of overlap among the enriched OTUs in each growth stage compared to the vegetative stage. Only six and five OTUs were shared by the flowering and fruiting stages, but no enriched OTUs were shared amongst the senescence, flowering, and fruiting stages. The depleted OTUs, however, had a higher overlap in the context of growth stage. There were 93, 92, and 21 depleted OTUs in common among the three stages in the rhizosphere, rhizoplane and endosphere; the OTUs that were depleted in the flowering and fruiting stages were all found in the senescence stage in the endosphere as well (Figure 8B).

#### 3.6.3. Growth Stage Effects on Fungal Community Structure

The fungal communities differed significantly among the growth stages in all the three compartments. The dissimilarities among growth stage (PERMANOVA R^2^ = 0.663 in the bulk soil, 0.755 in the rhizosphere, and 0.807 in the endosphere) were significantly larger than that of rhizocompartments (PERMANOVA R^2^ = 0.451 in the vegetative stage, 0.695 in the flowering stage, 0.398 in the fruiting stage, and 0.188 in the senescence stage) (Table 1). The PCoA and NMDS also revealed an apparent separation pattern of fungal communities by growth stage (Figure 3 and Figure 4), suggesting that the growth stage imposed a much stronger influence than the root compartments.

The changes in the community composition that were brought on by growth stage were also observed in each compartment. Ascomycota had highest abundance in the vegetative stage, and the lowest abundance in the fruiting stage, which is the opposite pattern than what Actinobacteria exhibited.

**Figure 9:**
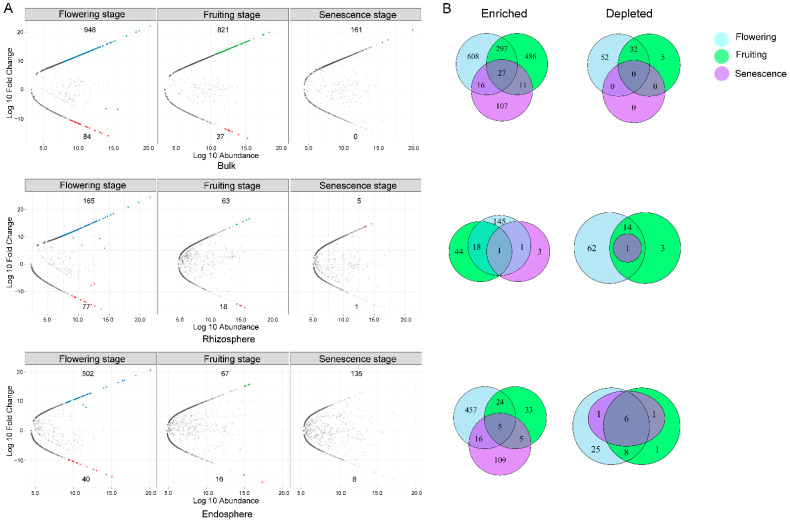
Fungal community comparison between the four growth stages in each root compartment. (**A**) MA diagrams show the enrichment and depletion of 18S OTUs for each growth stage (flowering stage, fruiting stage, and senescence stage) compared with the vegetative stage in three rhizocompartments. Each point represents an individual OTU, of which the blue, green, and purple points represent enriched OTUs in flowering stage, fruiting stage, and senescence stages compared to the vegetative stage, while the red ones indicate depleted OTUs, and the grey points represent no difference in abundance. The position along the y axis represents the abundance fold change compared with the bulk soil. (**B**) Venn diagrams illustrate the numbers of differentially enriched and depleted OTUs between each stage compared with the vegetative stage.

Compared to the vegetative stage, there were 165, 63, and 5 OTUs that were significantly enriched in the latter three growth stages in the rhizosphere. Even more OTUs were enriched in the endosphere from the flowering to senescence stages (502, 67, and 135 OTUs, respectively) (Figure 9A). However, only one and five enriched OTUs were shared in the rhizosphere and endosphere (Figure 9B). The five shared enriched OTUs in the endosphere belonged mainly to the species *Zopfiella marina*, *Saitozyma podzolica*, *Mortierella elongata*, and *Trechispora* sp.

There was also OTU exclusion in the different growth stages (Figure 9). There were 77 and 40 depleted OTUs in the rhizosphere and endosphere during the flowering stage. As plant development progressed, the exclusive OTUs numbers reduced; one and eight OTUs were depleted in the rhizosphere and endosphere, respectively, in the senescence stage. In the endosphere, six out of the eight depleted OTUs in the senescence stage were also found to be depleted in the flowering and fruiting stages, which were mainly affiliated with *Fusarium* (*F. nematophilum*), *Phomopsis*, *Roussoella*, *Aspergillus* (*A. clavatus*), and *Aporospora*.

### 3.7. Growth Stage-Specific Biomarkers Identification in Each Comparment

There were 56, 54, 52, and 47 bacterial biomarkers found in the bulk soil, rhizosphere, rhizoplane, and endosphere, respectively (Figures S15–S18). While 79, 84, and 87 fungal biomarkers were detected in the bulk soil, rhizosphere, and endosphere, respectively (Figures S19–S21).

A total of nine rhizospheric bacterial biomarkers were detected in the vegetative stage; the genera *Pseudomonas*, *Lysobacter*, and *Devosia* were more abundant in this time than in any other stage. Planctomycetia (*Pelagibius*, *Ensifer*, *Blastopirellula*, and *Roseovarius*) was more abundant in the flowering stage; Thermomicrobia, Actinobacteria, *Enterobacter*, and *Rhodoligotrophos* were significantly enriched in the fruiting stage; and Firmicutes (genera of *Bacillus*, *Planomicrobium*, *Salinimicrobium*, *Halomonas*, *Mesorhizobium*, *Deferrisoma*, and *Microbulbifer*) were more abundant in the senescence stage (Figure S16). Most of growth stage-specific bacterial biomarkers that were detected in the rhizosphere were also observed in the rhizoplane. For example, there were *Lysobacter* in the vegetative stage; *Ensifer* and *Roseovarius* in flowering stage; *Thermomicrobia* in the fruiting stage; and *Mesorhizobium*, *Halomonas*, *Microbulbifer*, *Bacillus*, and *Deferrisoma* in the senescence stage. This suggests a similarity between the rhizosphere and rhizoplane, but there were also differences that were found in the many more genera biomarkers that were present (i.e., *Pseudomonas*, *Brevundimonas*, *Weissella*, *Phenylobacterium*, *Aquamicrobium*, *Galbibacteri*) (Figure S17). Much fewer biomarkers were detected in the endosphere in the vegetative (*Labrenzia*, *Sphingopyxis*) and flowering (*Ensifer*, *Gracilimonas*) stages, but there were more that were found in the latter two stages. *Murinocardiopsis*, *Enterobacter*, *Chlorobium*, *Nitratireductor*, and *Acinetobacter* were more abundant in the fruiting stage, while *Galbibacter*, *Brevundimonas*, *Aquihabitans*, *Aquamicrobium*, *Pseudomonas*, and *Sphingorhabdus* dominated in the senescence stage (Figure S18).

The species diversity and composition of fungal biomarkers differed by growth stage in both the rhizosphere and endosphere. There were 36, 12, 19, and 17 clades (from phylum to species level) that were detected in rhizosphere, while the 32, 13, 9, and 33 were found in the endosphere. The least was detected in the fruiting stage (Figures S19–S21). In the rhizosphere, the discriminating clades were predominantly Ascomycota and Basidiomycota (*Udeniomyces puniceus*, *Vishniacozyma carnescens*, *Filobasidium*, and *Mycosphaerella tassiana*) in the vegetative stage. The fruiting stage was enriched with Rozellomycota (*Scedosporium prolificans*, *Sarocladium bactrocephalum*, *Lentinula edodes*, and *Simplicillium lanosoniveum*), and the senescence stage was enriched with Mortierellomycota (*Penicillium chrysogenum*, *Anthopsis*, *Mortierella*, *Coprinopsis clastophylla*, *Alternaria hlamydospore*, and *Corollospora maritima*) (Figure S20). Much like the rhizosphere, Ascomycota was most abundant in the endosphere in the vegetative stage, Rozellomycota was enriched in the fruiting stage, and Mortierellomycota and Basidiomycota were more abundant in the senescence stage (Figure S21).

## 4. Discussion

### 4.1. The Dominant Root-Associated Microbial Community Composition

The plant root is an important microhabitat for microbial colonization; a complex and dynamic microbiome arises, with significantly different community compositions between the rhizosphere, rhizoplane, and endosphere compartments [3,4]. In this study, we investigated the effect of growth stage on the dynamics of bacterial and fungal communities across the three root compartments and four growth stages. The dominant bacteria that were observed were Proteobacteria (Alpha-, Delta-, and Gamma-proteobacteria), Actinobacteria, and Bacteroidetes in both the bulk soil and the three rhizocompartments. Ascomycota and Basidiomycota were the most dominant fungal phyla that were found in most of the samples, however, a large proportion of the sequences (22.32% to 75.59%) were assigned to unclassified organisms and unclassified fungi, especially in the flowering to senescence stages.

### 4.2. Bacterial Community Differed between Root Compartments

We observed significant differences in the bacterial communities among the three compartments in both diversity and structure. The diversity and richness were highest in the rhizosphere, followed by the bulk soil and rhizoplane, and lowest in the endosphere, much like the results that were reported in rice [3]. Further, bacterial community structures differed greatly between rhizocompartments throughout the whole growing period. The WUF PCoA and NMDS indicated that the largest source of variation in root-associated bacterial communities was proximity to the root. PERMANOVA corroborates that rhizospheric compartmentalization comprises the largest source of variation.

The rhizosphere is the first interface for the root to recruit microbiomes; the roots specifically select microbial communities through rhizodeposition, thus cultivating microbiota that is generally different from the bulk soil [4,24,25]. Indeed, the rhizosphere had a higher content of TOC and TN and a lower relative pH value than what was found in the bulk soil during the whole growth cycle (Table S1). The effect of host selection was greater in the endosphere than in the rhizosphere, leading to a distinct and less diverse population in the endosphere compared to the rhizosphere [4]. The exclusivity in the endosphere relative to the rhizosphere was reported in other studies as well [2,3,4].

Similarly, we observed the dynamism of the dominant phyla across the bulk soil and the three compartments. Proteobacteria increased from the bulk soil to the rhizoplane and dropped slightly in the endosphere while they were still higher than in the bulk soil. Previous studies have shown that Proteobacteria were effective rhizosphere- and root-colonizers in several plants [3,26]. In contrast, the relative abundances of Actinobacteria decreased from the bulk soil to the rhizoplane, and then increased slightly in the endosphere while still higher than in the bulk soil, which aligns with previous studies in *Arabidopsis* [1,27]. Actinobacteria are also dominant in soils that were affected by salinity and drought and are usually more abundant in the endosphere of halophytes [28]. With their abundance and their ability to produce a large number of antimicrobial compounds and degrade a variety of toxic organic compounds [29,30], Actinobacteria have the potential to promote plant growth in hypersaline environments. Although Acidobacteria decreased in abundance from the soil to the endosphere, they have a large proportion of genes that encode for transporters, which can facilitate the acquisition of a broad range of substrate categories [31].

### 4.3. Differential Analysis Identified Root Compartment-Specific Enriched or Depleted Bacteria

The enrichment analysis showed that a part of the OTUs that are enriched in the rhizosphere are also enriched in the rhizoplane and/or endosphere. The enriched OTUs were mainly affiliated with 69 genera (Figures S3–S6). There were 24 out of 69 genera that appeared at least two growth stages, three (*Devosia*, *Pirellula*, and *Gimesia*) appeared at three stages, and seven (*Actinophytocola*, *Mycobacterium*, *Nocardia*, *Nocardioides*, *Arenicella*, *Haliea*, and *Porphyrobacter*) were present in all four stages. This suggests that plant roots play a role in enriching soil for specific bacteria with the growth cycle and its corresponding changes in plant metabolic activity, which can be observed where some bacteria are essential during the whole growth cycle, whereas some are unique to a specific stage of growth. We found that most of the enriched OTUs are not only halotolerant bacteria, but also have potential plant growth-promoting potential. Amongst many of the bacteria, *Porphyrobacter* can produce indole-3-acetic acid and solubilize phosphate [32]. *Actinophytocola* members have been isolated from the rhizosphere and endosphere of several plants [33,34], and play an important role in stress adaptation [35,36]. *Nocardia* were identified from the root endosphere of halophytic plants [28] and are important facilitators of phytoremediation of VOCs as they can remove toxic compounds from the soil [37]. Many *Nocardioides* species have the ability to degrade specific pollutants and can be applied in industry and agriculture [38]. In addition, similar to previous reports that found *Mycobacterium* in relatively high abundance in several tree plant endospheres [39], this genus could accelerate the degradation of organic contaminants (i.e., a variety of PAHs) and enhance plant resistance to soil pollution [40]. The prevalence of these potentially useful bacteria may be due to the host selecting for beneficial bacteria that can promote plant growth and confer resistance to environmental stress.

Other genera that were enriched in one or two growth stages may also have beneficial effects on the plant. Genera such as *Gemmatimonas*, *Mesorhizobium*, *Novosphingobium*, *Devosia*, and *Ensifer* might help maintain plant hormone balance, control root development, facilitate nutrition acquisition, and prevent disease in the host plant [41,42,43,44]. *Methylophaga*, *Microbulbifer*, *Sandaracinus*, *Haliangium*, and *Povalibacter* are found to be abundant or dominate in halophytic plant roots and are able to degrade starch and polysaccharides; oxidize methane, methanol, and hydrocarbons; or improve the salt tolerance of the host plant [45,46,47,48,49]. *Lysobacter* participate in N_2_ fixation and environmental pollutants degradation [41]. *Pirellula* have been found to have suppressive effects against parasitic nematodes [50].

Except for the enriched OTUs that are shared by all three root compartments, the rhizoplane or endosphere are uniquely enriched with a subset of OTUs (Figure 5 and Figure 6), which suggests that the rhizoplane and endosphere serve as a specialized niche for some taxa.

The number of depleted OTUs increased in all of the four growth stages. A large proportion of the depleted microbes in the rhizosphere are also depleted in the rhizoplane and endosphere, indicating that the selection for endophytic colonization begins at the rhizoplane, and that the rhizoplane may play a gating role by limiting microbial penetrance into the endosphere [3].

The enrichment and depletion of certain microbes across the rhizocompartments indicates that plants may have the ability to select for certain microbial consortia, or that some microbes are better at filling the specific niches for root colonization. Many studies have indicated that the plant can influence the root microbiota through rhizoexudations and select for beneficial microorganisms for colonization to promote growth, development, and stress tolerance in plants [11,12].

### 4.4. Root Compartment-Specific Bacterial Biomarkers Varied with Growth Stage

In addition to the differential analyses, biomarker identification was used to further support the differences that were found by the rhizocompartments. Across the growth cycle, the bacterial biomarkers in the rhizosphere and rhizoplane were fewer in the first three stages, but then rose in the senescence stage (33, 25, and 19 clades in the vegetative, flowering, and fruiting stages, respectively, vs. 52 clades in the senescence stage). Several genera were predominant and determined the dissimilarities among the compartments (Figures S7–S10). Many of the biomarkers, including for *Bacillus*, *Pseudomonas*, *Agrobacterium*, *Gemmatimonas*, *Enterobacter*, *Streptomyces*, *Devosia*, *Ensifer*, *Acinetobacter*, *Halomonas*, *Brevundimonas*, *Mesorhizobium*, and *Burkholderia*, are known as beneficial microbes for plants, and these microbes may help to maintain plant hormone balance, control root development, facilitate nutrition acquisition, and prevent disease in the plant host [42,44,51,52,53,54,55]. Several studies have reported that *Streptomycetes* are abundant inside of the roots of *Arabidopsis thaliana* [56] where they can have beneficial effects on growth [57] or protect plants against biotic and abiotic stressors [58,59].

### 4.5. Differential Analysis Identified Root Compartment-Specific Enriched or Depleted Fungi

The fungal community diversity and richness did not show a consistent trend across the four stages, but we found that there was a significant difference in the flowering stage. There was no significant difference in the richness and diversity between the four compartments in the fruiting and senescent stages, suggesting that fungal richness and diversity is stable across the root with the progression of development.

Relative to the bulk soil, there were only a few enriched or depleted OTUs in the rhizosphere or endosphere, and the endosphere exhibited higher exclusivity than the rhizosphere. The enriched and depleted OTUs were observed in the flowering stage, though the differential OTUs in comparison to the bulk soil was relatively lower in the other three stages, especially in the fruiting and senescence stages, which indicated a relative high similarity between the root-associated and bulk soil fungal communities. This is consistent with the ANOSIM and PERMANOVA analyses that showed there was no significant difference between the three spatial compartments in the latter two growth stages. Moreover, the fungal community in the PCoA and NMDS implied that the effect of the root compartment on the fungal community composition was weak.

Compared to the bulk soil, some fungal genera or species were enriched in the rhizosphere or endosphere, but only a part of them were enriched in both root compartments (Figure 6A, Tables S16–S19). Some of the fungi are beneficial, for instance, *Halosarpheia* (*H. japonic*), *Saitozyma podzolica*, *Zopfiella*, *Pestalotiopsis*, *Myrothecium,* and *Cladorrhinum* are involved in degrading macromolecular substances into smaller molecular substances that are easily utilized by cell metabolism [60]. Some species of *Trechispora* are ectomycorrhizal fungi and can provide water and nutrients (e.g., phosphorus or nitrogen) [61], help seed establishment and seedling development, and protect plants from root pathogens [62]. *Roussoella* and *Phomopsis* have a repressive effect on pathogenic fungi *Candida albicans* and *Fusarium oxysporum* [63]. Some *Mortierella* have the capacity to improve plant growth by producing antibiotics and phytohormones, enhancing soil nutrients, and assisting phosphorus acquisition [64,65,66].

More than half of the depleted OTUs in the rhizosphere were also excluded from the endosphere (Figure 6B, Tables S20–S22). Some of these depleted fungi may be harmful, such as *Metarhizium anisopliae* and *Fusarium* (i.e., *F. oxysporum*), which are potential entomopathogenic fungi, and can cause root rot and wilt in various crops [67,68]. This further supports that the plant can positively select for specific root-associated microbiomes by not only attracting desirable microbes, but also excluding some harmful species from the root microbiome. However, it should be noted that not all rhizospheric *Metarhizium* are harmful; they can promote plant growth [69], but are depleted from the root compartments for unknown reasons.

### 4.6. Root Compartment-Specific Fungal Biomarkers Varied with Growth Stage

Consistent with the differential analysis, the rhizosphere and endosphere compartments had fewer biomarkers than what was found in the bulk soil in the earlier stages. Many biomarkers were identified in the bulk soil, but the number reduced dramatically in the root compartments in the flowering stage, which is indicative of a strong filtering effect from the rhizosphere to the soil fungi, which corroborates our findings in bacteria [3]. The compartment-specific biomarkers varied with growth stage; the most fungal biomarkers were detected in the vegetative stage, and the fewest were detected in the senescence stage, which further suggests the stability of the fungal community structure throughout the plant growth cycle.

The potential role and function of fungi that was proposed in this study has been retrieved from the literature. Although we know the possible beneficial or harmful impact of some fungi on plants, there was a large proportion of unidentified fungi and, therefore, unknown function, found in the rhizosphere and endosphere. These unclassified fungi may have not-yet-discovered interactions with helpful or harmful organisms, or they may play a role in establishing beneficial symbioses with their plant hosts [3,4].

### 4.7. Growth Stage Dynamics of Micobiomes Diversity and Structure in Root Compartment

The stage of plant development is an important driver in shaping rhizospheric communities, leading to a dynamic exchange between plant growth and the host’s microbiota composition [4,70,71]. We found that the bacterial diversity and richness was the highest during the fruiting stage, although there was no significant difference found in the other three stages. In contrast to the high bacterial diversity of the summer samples, the senescent samples from the winter exhibited the lowest diversity [7]. The fungal community diversity and richness, however, showed that there were significant differences among the four stages; it was the lowest in the vegetative stage in the spring and grew higher in the summer when the plant was fruiting (Figure 7). Li et al. [7] reported that their summer sample displayed the highest fungal diversity as well, but the lowest was observed in the senescent stage in the winter. In contrast, the fungal richness and diversity of *Pinus tabuliformis* roots decreased during warm seasons [72]. Likewise, Liu et al. [71] observed the greatest arbuscular mycorrhizal richness and diversity in the spring for *Clematis fruticosa*. These observations not only further support the possibility that the season and/or plant development stage impacts the microbial community diversity and richness, but also that the specific effects could be varied due to the differences in species, and other biotic or abiotic factors.

The growth stage changes in both bacterial and fungal communities were identified by PERMANOVA, LefSe, and enrichment and depletion analysis. The growth stage was influential but explained only a small fraction of the bacterial variation (Table 1). The enriched or depleted bacterial OTUs in the flowering, fruiting, and senescence stages gradually increased in all the four compartments compared to the vegetative stage (Figure 8). This suggests that the enhanced filter effects that were imposed by the plant occurred through the growth cycle. In contrast to the rhizosphere and rhizoplane, the composition and structure of the bacterial community in the endosphere remained relatively stable from the vegetative to the fruiting stage and there were fewer significantly enriched or reduced OTUs. For fungi, the dissimilarities among the growth stages were higher (Table 1); in conjunction with the spatial cluster pattern (Figure 3 and Figure 4), suggested that the growth stage imposed a much stronger impact on fungal community composition than the root compartment.

In a given root compartment, the growth stage dynamics of bacterial and fungal composition and abundance was uncovered in *L. ruthenicum*. Additionally, the biomarkers also varied with the growth stage. Growth stage-specific bacterial biomarkers in the bulk soil, rhizosphere, rhizoplane, and endosphere soils were detected, and they differed among the four growth stages (Figures S15–S18). Moreover, a large part of these bacterial biomarkers have known beneficial effects on the plant host, such as those from *Pseudomonas*, *Lysobacter*, *Bacillus*, *Halomonas*, and *Mesorhizobium*. Similar to bacteria, growth stage-specific fungal biomarkers differed between the growth stages (Figures S19–S21), which showed that the flowering and fruiting stage in the summer harbored relatively fewer biomarkers in the warmest season. This corroborated with the findings in Li et al. [7] which reported that biomarkers in the rhizosphere were significantly affected by seasonal variation, and the most bacterial and fungal biomarkers were detected in winter, and the fewest in the summer.

### 4.8. Growth Stage Dynamics of Microbial Structure Might Be Induced by Plant Rhizodeposits and Abiotic Factors of Seasonal Changes

The dynamic progression of root-associated microbial consortia may be positively selected by plant activities (i.e., exudate content and composition that is released by roots) in concert with abiotic factors [73,74,75]. Indeed, previous studies have supported that rhizodeposits may heavily influence the assembly of root-associated microbiomes [76]. For example, the exudation pattern in *Arabidopsis thaliana* plants vary depending on its growth stage; sugars are the most exuded compounds in the early stages of development but shifts into amino acids and phenolics in older plants, thus affecting the establishment of microbial communities in the rhizosphere [77].

It is important to note that the host plant’s root exudation pattern can be affected quantitatively and qualitatively by various abiotic (e.g., drought, salinity, temperatures, or nutrient starvation) and biological factors (e.g., species, age, or growth stage) [75,78]. We found that the diversity and richness decreased from the fruiting stage in the summer to the senescence stage in the early winter in the bulk soil, rhizosphere, and rhizoplane, but not the endosphere. The soil temperature increased from the vegetative stage to the fruiting stage where it peaked, then decreased in the senescence stage. Simultaneously, the soil water content decreased from the vegetative to senescence stage, where it was the highest in the fruiting stage and the lowest in the vegetative stage (Table S1).

Changes in the rhizospheric bacterial communities with the context of plant age have also been reported in some agricultural crops [79,80]. However, we did not consider the effect of plant age on root-associated microbial community diversity and composition because the age of the sampled *L. ruthenicum* individuals were not the same. Still, there are some differences in the microbial community structure in each replicate as demonstrated by PcoA and NMDS analysis, although the samples from the same stage did not cluster tightly.

### 4.9. Difference Pattern between Bacteria and Fungi

The effects of growth stage or seasonality in determining the root-associated microbial community composition depends on the host species and/or the rhizocompartment [81]. This study showed that the growth stage influenced the microbial community structure between rhizocompartments. The total fungal community variation that was caused by growth stage increased from the rhizosphere to the endosphere. For bacterial communities, however, the effects were higher in the rhizosphere than in the endosphere. In studies on wild and cultivated agave species, the season was the greatest contributing factor to variance in the root endosphere microbiome, although the rhizosphere community was primarily influenced by the host species [82,83]. Similar heightened influence by season within the root endosphere was also seen in cacti [84] and *Populus deltoides* [85], which may reflect the increased plant–microbe interactions within the roots [75].

The dissimilarities in bacterial community structure among the growth stages were lower than what was observed between the rhizocompartments. Further, PCoA suggested that the bacterial communities differed significantly between the rhizocompartments rather than by growth stage. In contrast, PCoA and NMDS revealed that the differences in the fungal communities were more affected by the growth stage than by the rhizocompartments (Table 1). Li et al. [7] found that seasonal variation in the rhizospheric bacteria was nonsignificant, but significant in the rhizospheric fungi of the desert shrub *Camellia yuhsienensis*. These indicated that the rhizocompartment and growth stage both have effects on the root-associated microbial community structure, but the effects were different in bacteria and fungi, indicating a unique response between bacteria and fungi.

## 5. Conclusions

The bacterial community structure of *L. ruthenicum* exhibited significant differences between the three root compartments and the bulk soil, and the rhizocompartment explained a large proportion of the total bacterial variation. Each compartment was enriched for specific bacteria and had unique biomarkers. Moreover, the composition of enriched bacteria and biomarkers in each compartment altered with growth stage. For fungi, a significant rhizocompartment difference was only observed in the vegetative and flowering stages, but not in the fruiting and senescence stages. The growth stage explained a larger proportion of the total community variation than the rhizocompartment. Differential and biomarker analysis revealed that the fungal community composition in the rhizosphere and endosphere exhibited dynamic responses to the growth stage and imposed a much stronger impact than the root compartment on fungal community structures. Many enriched OTUs or biomarkers that were identified in the root compartments had been demonstrated to have beneficial effects on the plant, implying that the host plant positively selects for specific beneficial bacteria and fungi to promote plant growth or salt-tolerance in halophyte plants. The root compartment and growth stage were two important determinant factors in structuring the microbial communities of *L. ruthenicum*, but the effect was different in bacteria and fungi, indicating bacterial and fungal communities respond uniquely to the growth stage and rhizocompartment.

## Figures and Tables

**Table 1:** ANOSIM and PERMANOVA analysis between the rhizocompartments and growth stages of bacterial and fungal communities.

Microbial Community	Growth Stage	Rhizocompartment	PERMANOVA		ANOSIM (Genus Level)	
			Statistic (R^2^)	*p* Value	Statistic (R)	*p* Value
Bacteria/Archaea 16S	Between growth stages	Bulk	0.268	0.106	0.222	0.019
		Rhizosphere	0.554	0.002	0.568	0.001
		Rhizoplane	0.274	0.14	0.122	0.123
		Endosphere	0.339	0.047	0.247	0.021
	Between rhizocompartment	Vegetative	0.526	0.001	0.373	0.001
		flowering	0.527	0.001	0.456	0.001
		fruiting	0.637	0.001	0.483	0.001
		Senescence	0.637	0.001	0.449	0.001
Fungi 18S	Between growth stages	Bulk	0.663	0.001	0.717	0.001
		Rhizosphere	0.755	0.001	0.804	0.001
		Endosphere	0.807	0.001	0.852	0.001
	Between rhizocompartment	Vegetative	0.451	0.009	0.308	0.032
		flowering	0.695	0.007	0.361	0.012
		fruiting	0.398	0.027	0.144	0.079
		Senescence	0.188	0.718	−0.021	0.503

## Data Availability

The sequencing raw data have been deposited in NCBI under the accession number PRJNA843873.

## Supplementary Materials

The following supporting information can be downloaded at: https://www.mdpi.com/article/10.3390/microorganisms10081644/s1, Figure S1: Rarefaction curve of 16S rDNA showed that the number of OTUs increased with the number of sequences that were obtained in bulk soil, rhizosphere, rhizoplane, and endosphere samples. Figure S2: Rarefaction curve of 18S rDNA showed that the number of OTUs increased with the number of sequences that were obtained in bulk soil, rhizosphere, endosphere samples. Figure S3: The shared 16S OTUs that were enriched in rhizosphere, rhizoplane, and endosphere compared to bulk soil in the vegetative stage. Figure S4: The shared 16S OTUs that were enriched in rhizosphere, rhizoplane, and endosphere compared to bulk soil in the flowering stage. Figure S5: The shared 16S OTUs that were enriched in rhizosphere, rhizoplane, and endosphere compared to bulk soil in the fruiting stage. Figure S6: The shared 16S OTUs that were enriched in rhizosphere, rhizoplane, and endosphere compared to bulk soil in the senescence stage. Figure S7: LEfSe was used to identify rhizocompartment-specific (bulk soil, rhizosphere, rhizoplane, and endosphere) bacterial biomarkers in the vegetative stage. Cladogram that was generated by LEfSe indicating differences of bacteria (A) at the phylum, class, family, and genus levels between the four compartments (relative abundance ≤ 0.5%). Each successive circle represents a phylogenetic level. Color regions indicate taxa that are enriched in the different compartments. Differing taxa are listed on the right side of the cladogram. Bar graph showing the LDA scores for bacteria (B). Only taxa meeting an LDA significant threshold >3 are shown. Figure S8: LEfSe was used to identify rhizocompartment-specific (bulk soil, rhizosphere, rhizoplane, and endosphere) bacterial biomarkers in the flowering stage. Figure S9: LEfSe was used to identify rhizocompartment-specific (bulk soil, rhizosphere, rhizoplane, and endosphere) bacterial biomarkers in the fruiting stage. Cladogram that was generated by LEfSe indicating differences of bacteria (A) at phylum, class, family, and genus levels between the four compartments (relative abundance ≤ 0.5%). Each successive circle represents a phylogenetic level. Color regions indicate taxa that were enriched in the different compartments. Differing taxa are listed on the right side of the cladogram. Bar graph showing the LDA scores for bacteria (B). Only taxa meeting an LDA significant threshold >3 are shown. Figure S10: LEfSe was used to identify rhizocompartment-specific (bulk soil, rhizosphere, rhizoplane, and endosphere) bacterial biomarkers in the senescence stage. Cladogram that was generated by LEfSe indicating differences of bacteria (A) at the phylum, class, family, and genus levels between the four compartments (relative abundance ≤ 0.5%). Each successive circle represents a phylogenetic level. Color regions indicate taxa that were enriched in the different compartments. Differing taxa are listed on the right side of the cladogram. Bar graph showing LDA scores for bacteria (B). Only taxa meeting an LDA significant threshold >3 are shown. Figure S11: LEfSe was used to identify rhizocompartment-specific (bulk soil, rhizosphere, and endosphere) fungal biomarkers in the vegetative stage. Cladogram that was generated by LEfSe indicating differences of fungai (A) at phylum, class, family, genus, and species levels between the four compartments (relative abundance ≤ 0.5%). Each successive circle represents a phylogenetic level. Color regions indicate taxa that were enriched in the different compartments. Differing taxa are listed on the right side of the cladogram. Bar graph showing LDA scores for fungi (B). Only taxa meeting an LDA significant threshold >3 are shown. Figure S12: LEfSe was used to identify rhizocompartment-specific (bulk soil, rhizosphere, and endosphere) fungal biomarkers in flowering stage. Cladogram that was generated by LEfSe indicating differences of fungai (A) at phylum, class, family, genus, and species levels between the four compartments (relative abundance ≤ 0.5%). Each successive circle represents a phylogenetic level. Color regions indicate taxa that were enriched in the different compartments. Differing taxa are listed on the right side of the cladogram. Bar graph showing LDA scores for fungi (B). Only taxa meeting an LDA significant threshold >3 are shown. Figure S13: LEfSe was used to identify rhizocompartment-specific (bulk soil, rhizosphere, and endosphere) fungal biomarkers in the fruiting stage. Cladogram that was generated by LEfSe indicating differences of fungi (A) at the phylum, class, family, genus, and species levels between the four compartments (relative abundance ≤ 0.5%). Each successive circle represents a phylogenetic level. Color regions indicate taxa that were enriched in the different compartments. Differing taxa are listed on the right side of the cladogram. Bar graph showing LDA scores for fungi (B). Only taxa meeting an LDA significant threshold >3 are shown. Figure S14: LEfSe was used to identify rhizocompartment-specific (bulk soil, rhizosphere, and endosphere) fungal biomarkers in the senescence stage. Cladogram that was generated by LEfSe indicating differences of fungi (A) at the phylum, class, family, genus, and species levels between the four compartments (relative abundance ≤ 0.5%). Each successive circle represents a phylogenetic level. Color regions indicate taxa that were enriched in the different compartments. Differing taxa are listed on the right side of the cladogram. Bar graph showing LDA scores for fungi (B). Only taxa meeting an LDA significant threshold >3 are shown. Figure S15: LEfSe was used to detect growth stage-specific bacterial biomarkers in bulk soil communities. Cladogram that was generated by LEfSe indicating differences of bacteria (A) at the phylum, class, family, and genus levels between the four growth stages (relative abundance ≤ 0.5%). Each successive circle represents a phylogenetic level. Color regions indicate taxa that were enriched in the different growth stages. Differing taxa are listed on the right side of the cladogram. Bar graph showing LDA scores for bacteria (B). Only taxa meeting an LDA significant threshold >3 are shown. Figure S16: LEfSe was used to detect growth stage-specific bacterial biomarkers in rhizospheric soil communities. Cladogram that was generated by LEfSe indicating differences of bacteria (A) at phylum, class, family, and genus levels between the four growth stages (relative abundance ≤ 0.5%). Each successive circle represents a phylogenetic level. Color regions indicate taxa that were enriched in the different growth stages. Differing taxa are listed on the right side of the cladogram. Bar graph showing LDA scores for bacteria (B). Only taxa meeting an LDA significant threshold >3 are shown. Figure S17: LEfSe was used to detect growth stage-specific bacterial biomarkers in rhizoplane soil communities. Cladogram that was generated by LEfSe indicating differences of bacteria (A) at the phylum, class, family, and genus levels between the four growth stages (relative abundance ≤ 0.5%). Each successive circle represents a phylogenetic level. Color regions indicate taxa that were enriched in the different growth stages. Differing taxa are listed on the right side of the cladogram. Bar graph showing LDA scores for bacteria (B). Only taxa meeting an LDA significant threshold >3 are shown. Figure S18: LEfSe was used to detect growth stage-specific bacterial biomarkers in endospheric communities. Cladogram that was generated by LEfSe indicating differences of bacteria (A) at the phylum, class, family, genus, and species levels between the four growth stages (relative abundance ≤ 0.5%). Each successive circle represents a phylogenetic level. Color regions indicate taxa that were enriched in the different growth stages. Differing taxa are listed on the right side of the cladogram. Bar graph showing LDA scores for bacteria (B). Only taxa meeting an LDA significant threshold >3 are shown. Figure S19: LEfSe was used to detect growth stage-specific fungal biomarkers in bulk soil communities. Cladogram that was generated by LEfSe indicating differences of fungi (A) at the phylum, class, family, genus, and species levels between the four growth stages (relative abundance ≤ 0.5%). Each successive circle represents a phylogenetic level. Color regions indicate taxa that were enriched in the different growth stages. Differing taxa are listed on the right side of the cladogram. Bar graph showing LDA scores for bacteria (B). Only taxa meeting an LDA significant threshold >3 are shown. Figure S20: LEfSe was used to detect growth stage-specific fungal biomarkers in rhizospheric soil communities. Cladogram that was generated by LEfSe indicating differences of fungi (A) at the phylum, class, family, genus, and species levels between the four growth stages (relative abundance ≤ 0.5%). Each successive circle represents a phylogenetic level. Color regions indicate taxa that were enriched in the different growth stages. Differing taxa are listed on the right side of the cladogram. Bar graph showing LDA scores for bacteria (B). Only taxa meeting an LDA significant threshold >3 are shown. Figure S21: LEfSe was used to detect growth stage-specific fungal biomarkers in endospheric communities. Cladogram that was generated by LEfSe indicating differences of fungi (A) at the phylum, class, family, genus, and species levels between the four growth stages (relative abundance ≤ 0.5%). Each successive circle represents a phylogenetic level. Color regions indicate taxa that were enriched in the different growth stages. Differing taxa are listed on the right side of the cladogram. Bar graph showing LDA scores for bacteria (B). Only taxa meeting an LDA significant threshold >3 are shown. Table S1: Soil physico-chemical properties of the bulk and rhizosphere soils in different growth stages. Table S2: Statistics of 16S rDNA sequencing data and diversity indices of samples. Table S3: Statistics of 18S rDNA sequencing data and diversity indices of samples. Table S4: 16S rDNA OTU abundance and taxonomy in each sample. Table S5: 18S rDNA OTU abundance and taxonomy in each sample. Table S6: Bacterial and Archaeal community composition and relative abundance in each sample at the phylum and genus level. Table S7: Fungal community composition and relative abundance in each sample at the phylum and genus level. Table S8: The shared 16S OTUs that were enriched in the rhizosphere, rhizoplane, and endosphere compared to bulk soil in the vegetative stage. Table S9: The shared 16S OTUs that were enriched in the rhizosphere, rhizoplane, and endosphere compared to bulk soil in the flowering stage. Table S10: The shared 16S OTUs that were enriched in the rhizosphere, rhizoplane, and endosphere compared to bulk soil in the fruiting stage. Table S11: The shared 16S OTUs that were enriched in the rhizosphere, rhizoplane, and endosphere compared to bulk soil in the senescence stage. Table S12: The shared 16S OTUs that were depleted in the rhizosphere, rhizoplane, and endosphere compared to bulk soil in the vegetative stage. Table S13: The shared 16S OTUs that were depleted in the rhizosphere, rhizoplane, and endosphere compared to bulk soil in the flowering stage. Table S14: The shared OTUs that were depleted in the rhizosphere, rhizoplane, and endosphere compared to bulk soil in the fruiting stage. Table S15: The shared OTUs that were depleted in the rhizosphere, rhizoplane, and endosphere compared to bulk soil in senescence stage. Table S16: The shared 18S OTUs that were enriched in the rhizosphere and endosphere compared to bulk soil in the vegetative stage. Table S17: The shared 18S OTUs that were enriched in the rhizosphere and endosphere compared to bulk soil in the flowering stage. Table S18: The shared 18S OTUs that were enriched in the rhizosphere and endosphere compared to bulk soil in the fruiting stage. Table S19: The shared 18S OTUs that were enriched in the rhizosphere and endosphere compared to bulk soil in the senescence stage. Table S20: The shared OTUs that were depleted in the rhizosphere and endosphere compared to bulk soil in the vegetative stage. Table S21: The shared outs that were depleted in the rhizosphere and endosphere compared to bulk soil in the flowering stage. Table S22: The shared OTUs that were depleted in the rhizosphere and endosphere compared to bulk soil in the fruiting stage.

## References

[1] Bulgarelli D., Rott M., Schlaeppi K., van Themaat E.V.L., Ahmadinejad N., Assenza F., Rauf P., Huettel B., Reinhardt R., Schmelzer E. (2012). Revealing structure and assembly cues for *Arabidopsis* root-inhabiting bacterial microbiota. Nature.

[2] Lundberg D.S., Lebeis S.L., Paredes S.H., Yourstone S., Gehring J., Malfatti S., Tremblay J., Engelbrektson A., Kunin V., del Rio T.G. (2012). Defining the core *Arabidopsis thaliana* root microbiome. Nature.

[3] Edwards J., Johnson C., Santos-Medellin C., Lurie E., Podishetty N.K., Bhatnagar S., Eisen J.A., Sundaresan V. (2015). Structure, variation, and assembly of the root-associated microbiomes of rice. Proc. Natl. Acad. Sci. USA.

[4] Edwards J.A., Santos-Medellin C.M., Liechty Z.S., Nguyen B., Lurie E., Eason S., Phillips G., Sundaresan V. (2018). Compositional shifts in root-associated bacterial and archaeal microbiota track the plant life cycle in field-grown rice. PLoS Biol..

[5] Gao Y.K., Cui J.H., Ren G.Z., Wei S.L., Yang P.Y., Yin C.P., Liang H.K., Chang J.H. (2021). Changes in the root-associated bacteria of sorghum are driven by the combined effects of salt and sorghum development. Environ. Microbiome.

[6] Goodrich J.K., Davenport E.R., Waters J.L., Clark A.G., Ley R.E. (2016). Cross-species comparisons of host genetic associations with the microbiome. Science.

[7] Li J., Luo Z.Q., Zhang C.H., Qu X.J., Chen M., Song T., Yuan J. (2020). Seasonal variation in the rhizosphere and non-rhizosphere microbial community structures and functions of *Camellia yuhsienensis* Hu. Microorganisms.

[8] Knoth J.L., Kim S.H., Ettl G.J., Doty S.L. (2013). Effects of cross host species inoculation of nitrogen-fixing endophytes on growth and leaf physiology of maize. GCB Bioenergy.

[9] Egamberdieva D., Wirth S.J., Alqarawi A.A., Abd Allah E.F., Hashem A. (2017). Phytohormones and beneficial microbes: Essential components for plants to balance stress and fitness. Front. Microbiol..

[10] Qu Q., Zhang Z.Y., Peijnenburg W.J.G.M., Liu W.Y., Lu T., Hu B.L., Chen J.M., Chen J., Lin Z.F., Qian H.F. (2020). Rhizosphere microbiome assembly and its impact on plant growth. J. Agric. Food Chem..

[11] Mathur P., Roy S. (2021). Insights into the plant responses to drought and decoding the potential of root associated microbiome for inducing drought tolerance. Physiol. Plant..

[12] Roy S., Chakraborty A.P., Chakraborty R. (2021). Understanding the potential of root microbiome influencing salt-tolerance in plants and mechanisms involved at the transcriptional and translational level. Physiol. Plant..

[13] Dong L.L., Cheng R.Y., Xiao L.N., Wei F.G., Wei G.F., Xu J., Wang Y., Guo X.T., Chen Z.J., Chen S.L. (2018). Diversity and composition of bacterial endophytes among plant parts of *Panax notoginseng*. Chin. Med..

[14] Ibekwe A.M., Ors S., Ferreira J.F.S., Liu X., Suarez D.L. (2017). Seasonal induced changes in spinach rhizosphere microbial community structure with varying salinity and drought. Sci. Total Environ..

[15] Wu Z.Y., Lin W.X., Li J.J., Liu J.F., Li B.L., Wu L.K., Fang C.X., Zhang Z.X. (2016). Effects of seasonal variations on soil microbial community composition of two typical zonal vegetation types in the Wuyi Mountains. J. Mt. Sci..

[16] Peng F., Huang C.H., You Q.G., Gao T.P. (2013). Effects of plantation of *Lycium ruthenicum* on the soil salt distribution in the minqin basin. J. Desert Res..

[17] Schmieder R., Edwards R. (2011). Quality control and preprocessing of metagenomic datasets. Bioinformatics.

[18] Zhang J.J., Kobert K., Flouri T., Stamatakis A. (2014). PEAR: A fast and accurate Illumina Paired-End reAd mergeR. Bioinformatics.

[19] Edgar R.C., Haas B.J., Clemente J.C., Quince C., Knight R. (2011). UCHIME improves sensitivity and speed of chimera detection. Bioinformatics.

[20] Quast C., Pruesse E., Yilmaz P., Gerken J., Schweer T., Yarza P., Peplies J., Glockner F.O. (2013). The SILVA ribosomal RNA gene database project: Improved data processing and web-based tools. Nucleic Acids Res..

[21] Schloss P.D., Westcott S.L., Ryabin T., Hall J.R., Hartmann M., Hollister E.B., Lesniewski R.A., Oakley B.B., Parks D.H., Robinson C.J. (2009). Introducing mothur: Open-Source, Platform-Independent, Community-supported software for describing and comparing microbial communities. Appl. Environ. Microb..

[22] Love M.I., Huber W., Anders S. (2014). Moderated estimation of fold change and dispersion for RNA-seq data with DESeq2. Genome Biol..

[23] Segata N., Izard J., Waldron L., Gevers D., Miropolsky L., Garrett W.S., Huttenhower C. (2011). Metagenomic biomarker discovery and explanation. Genome Biol..

[24] Moriwaki T., Miyazawa Y., Kobayashi A., Uchida M., Watanabe C., Fujii N., Takahashi H. (2011). Hormonal regulation of lateral root development in *Arabidopsis* modulated by MIZ1 and requirement of GNOM activity for MIZ1 function. Plant Physiol..

[25] Umezawa T., Sugiyama N., Mizoguchi M., Hayashi S., Myouga F., Yamaguchi-Shinozaki K., Ishihama Y., Hirayama T., Shinozaki K. (2009). Type 2C protein phosphatases directly regulate abscisic acid-activated protein kinases in *Arabidopsis*. Proc. Natl. Acad. Sci. USA.

[26] Ai C., Liang G.Q., Sun J.W., Wang X.B., He P., Zhou W., He X.H. (2015). Reduced dependence of rhizosphere microbiome on plant-derived carbon in 32-year long-term inorganic and organic fertilized soils. Soil Biol. Biochem..

[27] Schlaeppi K., Dombrowski N., Oter R.G., van Themaat E.V.L., Schulze-Lefert P. (2014). Quantitative divergence of the bacterial root microbiota in *Arabidopsis thaliana* relatives. Proc. Natl. Acad. Sci. USA.

[28] Mukhtar S., Mehnaz S., Malik K.A. (2021). Comparative study of the rhizosphere and root endosphere microbiomes of Cholistan desert plants. Front. Microbiol..

[29] Deng S.S., Chang X.L., Zhang Y.M., Ren L.Z., Jiang F., Qu Z.H., Peng F. (2015). *Nocardioides antarcticus* sp nova, isolated from marine sediment. Int. J. Syst. Evol. Micr..

[30] Zhang H., Wang Z.F., Zhang Y.L., Ding M.J., Li L.H. (2015). Identification of traffic-related metals and the effects of different environments on their enrichment in roadside soils along the Qinghai-Tibet highway. Sci. Total Environ..

[31] Kielak A.M., Cipriano M.A.P., Kuramae E.E. (2016). Acidobacteria strains from subdivision 1 act as plant growth-promoting bacteria. Arch. Microbiol..

[32] Tahir M., Mirza M.S., Hameed S., Dimitrov M.R., Smidt H. (2015). Cultivation-based and molecular assessment of bacterial diversity in the rhizosheath of wheat under different crop rotations. PLoS ONE.

[33] Cao C.L., Sun Y., Wu B., Zhao S., Yuan B., Qin S., Jiang J.H., Huang Y. (2018). *Actinophytocola glycyrrhizae sp* nov isolated from the rhizosphere of Glycyrrhiza inflata. Int. J. Syst. Evol. Micr..

[34] Wang W., Wang B., Meng H.Y., Xing Z.B., Lai Q.L., Yuan L.J. (2017). *Actinophytocola xanthii* sp nov.; an actinomycete isolated from rhizosphere soil of the plant *Xanthium sibiricum*. Int. J. Syst. Evol. Microbiol..

[35] Cabanás C.G.L., Fernández-González A.J., Cardoni M., Valverde-Corredor A., López-Cepero J., Fernández-López M., Mercado-Blanco J. (2021). The Banana root endophytome: Differences between mother plants and suckers and evaluation of selected bacteria to control *Fusarium oxysporum* f. sp. Cubense. J. Fungi.

[36] Fernández-González A.J., Ramirez-Tejero J.A., Nevado-Berzosa M.P., Luque F., Fernández-López M., Mercado-Blanco J. (2021). Coupling the endophytic microbiome with the host transcriptome in olive roots. Comput. Struct. Biotechnol. J..

[37] McGuinness M., Dowling D. (2009). Plant-associated bacterial degradation of toxic organic compounds in soil. Int. J. Environ. Res. Public Health.

[38] Kim M.K., Srinivasan S., Park M.J., Sathiyaraj G., Kim Y.J., Yang D.C. (2009). *Nocardioides humi* sp nov.; A beta-glucosidase-producing bacterium isolated from soil of a ginseng field. Int. J. Syst. Evol. Microbiol..

[39] King W.L., Yates C.F., Guo J., Fleishman S.M., Trexler R.V., Centinari M., Bell T.H., Eissenstat D.M. (2021). The hierarchy of root branching order determines bacterial composition, microbial carrying capacity and microbial filtering. Commun. Biol..

[40] Dai Y.Y., Liu R., Zhou Y.M., Li N., Hou L.Q., Ma Q., Gao B. (2020). Fire Phoenix facilitates phytoremediation of PAH-Cd co-contaminated soil through promotion of beneficial rhizosphere bacterial communities. Environ. Int..

[41] Hu H.Y., Li H., Hao M.M., Ren Y.N., Zhang M.K., Liu R.Y., Zhang Y., Li G., Chen J.S., Ning T.Y. (2021). Nitrogen fixation and crop productivity enhancements co-driven by intercrop root exudates and key rhizosphere bacteria. J. Appl. Ecol..

[42] Jin K.M., Li H.B., Li X.Q., Li H.G., Dodd I.C., Belimov A.A., Davies W.J., Shen J.B. (2021). Rhizosphere bacteria containing ACC deaminase decrease root ethylene emission and improve maize root growth with localized nutrient supply. Food Energy Secur..

[43] Lemanceau P., Blouin M., Muller D., Moenne-Loccoz Y. (2017). Let the core microbiota be functional. Trends Plant Sci..

[44] Xu J., Zhang Y., Zhang P.F., Trivedi P., Riera N., Wang Y.Y., Liu X., Fan G.Y., Tang J.L., Coletta H.D. (2018). The structure and function of the global citrus rhizosphere microbiome. Nat. Commun..

[45] Chaluvadi S., Bennetzen J.L. (2018). Species-associated differences in the below-ground microbiomes of wild and domesticated Setaria. Front. Plant Sci..

[46] Li M., Jain S., Baker B.J., Taylor C., Dick G.J. (2014). Novel hydrocarbon monooxygenase genes in the metatranscriptome of a natural deep-sea hydrocarbon plume. Environ. Microbiol..

[47] Maarastawi S.A., Frindte K., Geer R., Krober E., Knief C. (2018). Temporal dynamics and compartment specific rice straw degradation in bulk soil and the rhizosphere of maize. Soil Biol. Biochem..

[48] Yaish M.W., Al-Lawati A., Jana G.A., Patankar H.V., Glick B.R. (2016). Impact of soil salinity on the structure of the bacterial endophytic community identified from the roots of caliph medic (*Medicago truncatula*). PLoS ONE.

[49] Yang H., Hu J.X., Long X.H., Liu Z.P., Rengel Z. (2016). Salinity altered root distribution and increased diversity of bacterial communities in the rhizosphere soil of *Jerusalem artichoke*. Sci. Rep..

[50] Engelbrecht G., Claassens S., Mienie C.M.S., Fourie H. (2021). Screening of rhizosphere bacteria and nematode populations associated with soybean roots in the Mpumalanga Highveld of South Africa. Microorganisms.

[51] Ferreira S.D., Nakasone A.K., do Nascimento S.M.C., de Oliveira D.A., Siqueira A.S., Cunha E.F.M., de Castro G.L.S., de Souza C.R.B. (2021). Isolation and characterization of cassava root endophytic bacteria with the ability to promote plant growth and control the in vitro and in vivo growth of *Phytopythium* sp.. Physiol. Mol. Plant Prod..

[52] Kang S.M., Joo G.J., Hamayun M., Na C.I., Shin D.H., Kim H.Y., Hong J.K., Lee I.J. (2009). Gibberellin production and phosphate solubilization by newly isolated strain of *Acinetobacter calcoaceticus* and its effect on plant growth. Biotechnol. Lett..

[53] Moslehi S., Pourmehr S., Shirzad A., Khakvar R. (2021). Potential of some endophytic bacteria in biological control of root-knot nematode *Meloidogyne incognita*. Egypt. J. Biol. Pest Control.

[54] Singh K., Gera R., Sharma R., Maithani D., Chandra D., Bhat M.A., Kumar R., Bhatt P. (2021). Mechanism and application of Sesbania root-nodulating bacteria: An alternative for chemical fertilizers and sustainable development. Arch. Microbiol..

[55] Vives-Peris V., de Ollas C., Gómez-Cadenas A., Pérez-Clemente R.M. (2020). Root exudates: From plant to rhizosphere and beyond. Plant Cell Rep..

[56] Bai Y.N., Eijsink V.G.H., Kielak A.M., van Veen J.A., de Boer W. (2016). Genomic comparison of chitinolytic enzyme systems from terrestrial and aquatic bacteria. Environ. Microbiol..

[57] Worsley S.F., Newitt J., Rassbach J., Batey S.F.D., Holmes N.A., Murrell J.C., Wilkinson B., Hutchings M.I. (2020). *Streptomyces* endophytes promote host health and enhance growth across plant species. Appl. Environ. Microbiol..

[58] Worsley S.F., Macey M., Prudence S., Wilkinson B., Murrell J.C., Hutchings M.I. (2021). Investigating the role of root exudates in recruiting streptomyces bacteria to the *Arabidopsis thaliana* microbiome. Front. Mol. Biosci..

[59] Newitt J.T., Prudence S.M.M., Hutchings M.I., Worsley S.F. (2019). Biocontrol of cereal crop diseases using Streptomycetes. Pathogens.

[60] Aliyu I.A., Yusuf A.A., Uyovbisere E.O., Masso C., Sanders I.R. (2019). Effect of co-application of phosphorus fertilizer and in vitro-produced mycorrhizal fungal inoculants on yield and leaf nutrient concentration of cassava. PLoS ONE.

[61] van der Heijden M.G.A., Martin F.M., Selosse M.A., Sanders I.R. (2015). Mycorrhizal ecology and evolution: The past, the present, and the future. New Phytol..

[62] Vanegas-León M.L., Sulzbacher M.A., Rinaldi A.C., Roy M., Selosse M.A., Neves M.A. (2019). Are Trechisporales ectomycorrhizal or non-mycorrhizal root endophytes?. Mycol. Prog..

[63] Huang Z.J., Cai X.L., Shao C.L., She Z.G., Xia X.K., Chen Y.G., Yang J.X., Zhou S.N., Lin Y.C. (2008). Chemistry and weak antimicrobial activities of phomopsins produced by mangrove endophytic fungus *Phomopsis* sp. ZSU-H76. Phytochemistry.

[64] Li F., Chen L., Redmile-Gordon M., Zhang J.B., Zhang C.Z., Ning Q., Li W. (2018). *Mortierella elongata*’s roles in organic agriculture and crop growth promotion in a mineral soil. Land Degrad. Dev..

[65] Tamayo-Velez A., Osorio N.W. (2018). Soil fertility improvement by litter decomposition and inoculation with the fungus *Mortierella sp* in avocado plantations of Colombia. Commun. Soil Sci. Plan..

[66] Zhang H.S., Wu X.H., Li G., Qin P. (2011). Interactions between arbuscular mycorrhizal fungi and phosphate-solubilizing fungus (*Mortierella sp.*) and their effects on *Kostelelzkya virginica* growth and enzyme activities of rhizosphere and bulk soils at different salinities. Biol. Fert. Soils.

[67] Qiu R., Bai J.K., Li C.J., Li S.J., Li X.J., Chen Y.G., Hu Y.J., Liu D.S. (2018). Molecular identification and pathogenicity analysis of tobacco *Fusarium* spp. in Henan. Acta Tab. Sin..

[68] Schlatter D., Kinkel L., Thomashow L., Weller D., Paulitz T. (2017). Disease suppressive soils: New insights from the soil microbiome. Phytopathology.

[69] Behie S.W., Bidochka M.J. (2014). Nutrient transfer in plant-fungal symbioses. Trends Plant Sci..

[70] Bencherif K., Boutekrabt A., Dalpe Y., Sahraoui A.L.H. (2016). Soil and seasons affect arbuscular mycorrhizal fungi associated with *Tamarix* rhizosphere in arid and semi-arid steppes. Appl. Soil Ecol..

[71] Liu M., Yue Y.J., Wang Z.H., Li L., Duan G.Z., Bai S.L., Li T. (2020). Composition of the arbuscular mycorrhizal fungal community and changes in diversity of the rhizosphere of *Clematis fruticosa* over three seasons across different elevations. Eur. J. Soil Sci..

[72] Wang H.H., Chu H.L., Dou Q., Feng H., Tang M., Zhang S.X., Wang C.Y. (2021). Seasonal changes in *Pinus tabuliformis* root-associated fungal microbiota drive N and P cycling in terrestrial ecosystem. Front. Microbiol..

[73] Buckeridge K.M., Banerjee S., Siciliano S.D., Grogan P. (2013). The seasonal pattern of soil microbial community structure in mesic low arctic tundra. Soil Biol. Biochem..

[74] Carbone M.J., Alaniz S., Mondino P., Gelabert M., Eichmeier A., Tekielska D., Bujanda R., Gramaje D. (2021). Drought influences fungal community dynamics in the grapevine rhizosphere and root microbiome. J. Fungi.

[75] Naylor D., Coleman-Derr D. (2018). Drought stress and root-associated bacterial communities. Front. Plant Sci..

[76] Cantó C.D., Simonin M., King E., Moulin L., Bennett M.J., Castrillo G., Laplaze L. (2020). An extended root phenotype: The rhizosphere, its formation and impacts on plant fitness. Plant J..

[77] Chaparro J.M., Badri D.V., Bakker M.G., Sugiyama A., Manter D.K., Vivanco J.M. (2013). Root exudation of phytochemicals in *Arabidopsis* follows specific patterns that are developmentally programmed and correlate with soil microbial functions. PLoS ONE.

[78] Williams A., de Vries F.T. (2020). Plant root exudation under drought: Implications for ecosystem functioning. New Phytol..

[79] Sugiyama A., Ueda Y., Takase H., Yazaki K. (2014). Pyrosequencing assessment of rhizosphere fungal communities from a soybean field. Can. J. Microbiol..

[80] Zimudzi J., van der Waals J.E., Coutinho T.A., Cowan D.A., Valverde A. (2018). Temporal shifts of fungal communities in the rhizosphere and on tubers in potato fields. Fungal Biol..

[81] Taketani R.G., Lanconi M.D., Kavamura V.N., Durrer A., Andreote F.D., Melo I.S. (2017). Dry season constrains bacterial phylogenetic diversity in a semi-arid rhizosphere system. Microb. Ecol..

[82] Coleman-Derr D., Desgarennes D., Fonseca-Garcia C., Gross S., Clingenpeel S., Woyke T., North G., Visel A., Partida-Martinez L.P., Tringe S.G. (2016). Plant compartment and biogeography affect microbiome composition in cultivated and native *Agave* species. New Phytol..

[83] Desgarennes D., Garrido E., Torres-Gomez M.J., Pena-Cabriales J.J., Partida-Martinez L.P. (2014). Diazotrophic potential among bacterial communities associated with wild and cultivated *Agave* species. FEMS Microbiol. Ecol..

[84] Fonseca-Garcia C., Coleman-Derr D., Garrido E., Visel A., Tringe S.G., Partida-Martinez L.P. (2016). The cacti microbiome: Interplay between habitat-filtering and host-specificity. Front. Microbiol..

[85] Shakya M., Gottel N., Castro H., Yang Z.M.K., Gunter L., Labbe J., Muchero W., Bonito G., Vilgalys R., Tuskan G. (2013). A multifactor analysis of fungal and bacterial community structure in the root microbiome of mature *Populus deltoides* trees. PLoS ONE.

