# Supplementary figures and images for: Differed Growth Stage Dynamics of Root-Associated Bacterial and Fungal Community Structure Associated with Halophytic Plant Lycium ruthenicum
Source: Microorganisms. 2022 Aug 15;10(8):1644. doi: 10.3390/microorganisms10081644 (PMC9414475; doi:10.3390/microorganisms10081644)

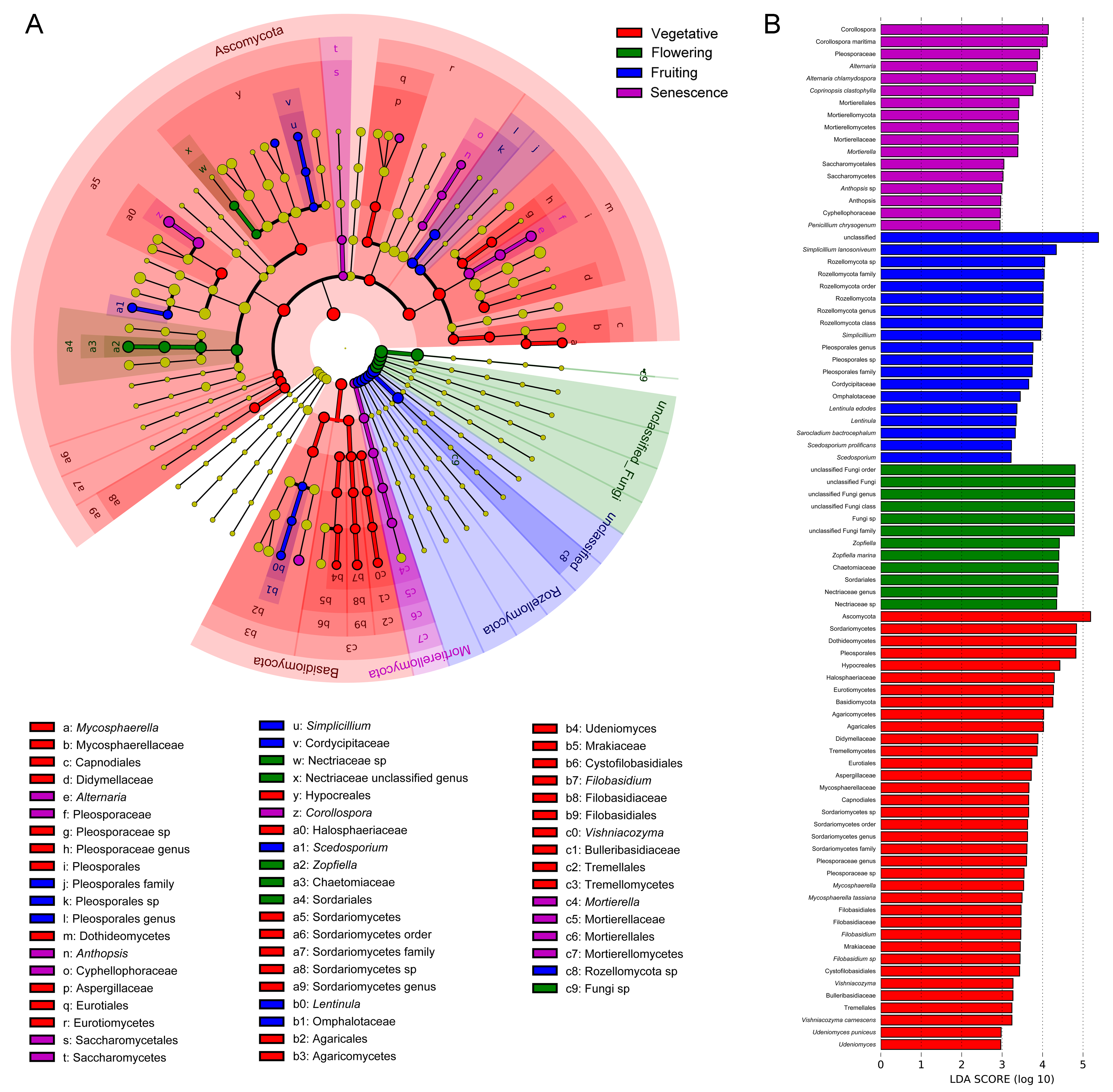

Supplement: Supplementary file 1 [file microorganisms-10-01644-s001.zip › Figure S20. LEfSe was used to detect growth stage-specific fungal biomarkers in rhizospheric soil communi-ties.tif]

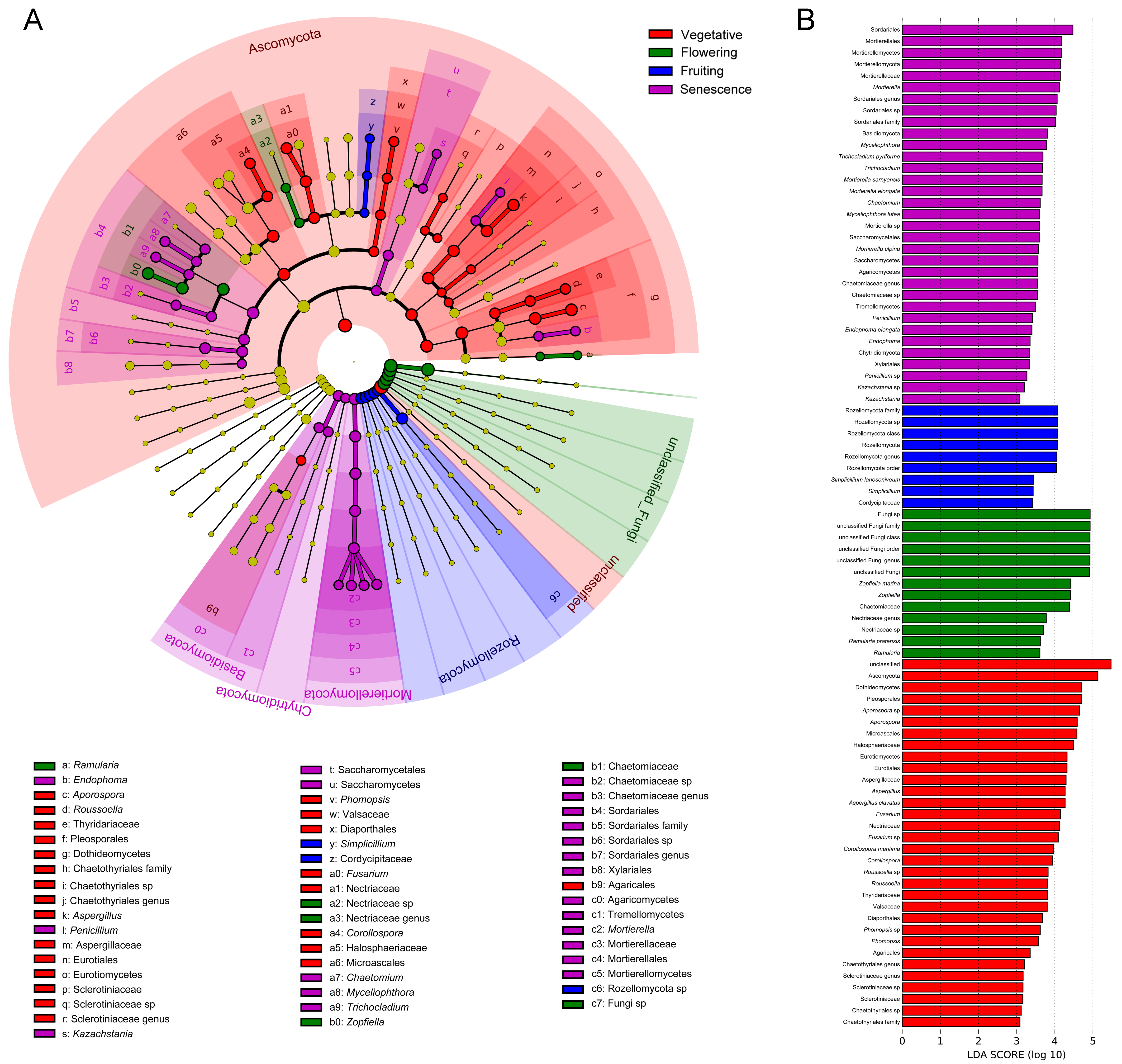

Supplement: Supplementary file 1 [file microorganisms-10-01644-s001.zip › Figure S21. LEfSe was used to detect growth stage-specific fungal biomarkers in endospheric communities.tif]

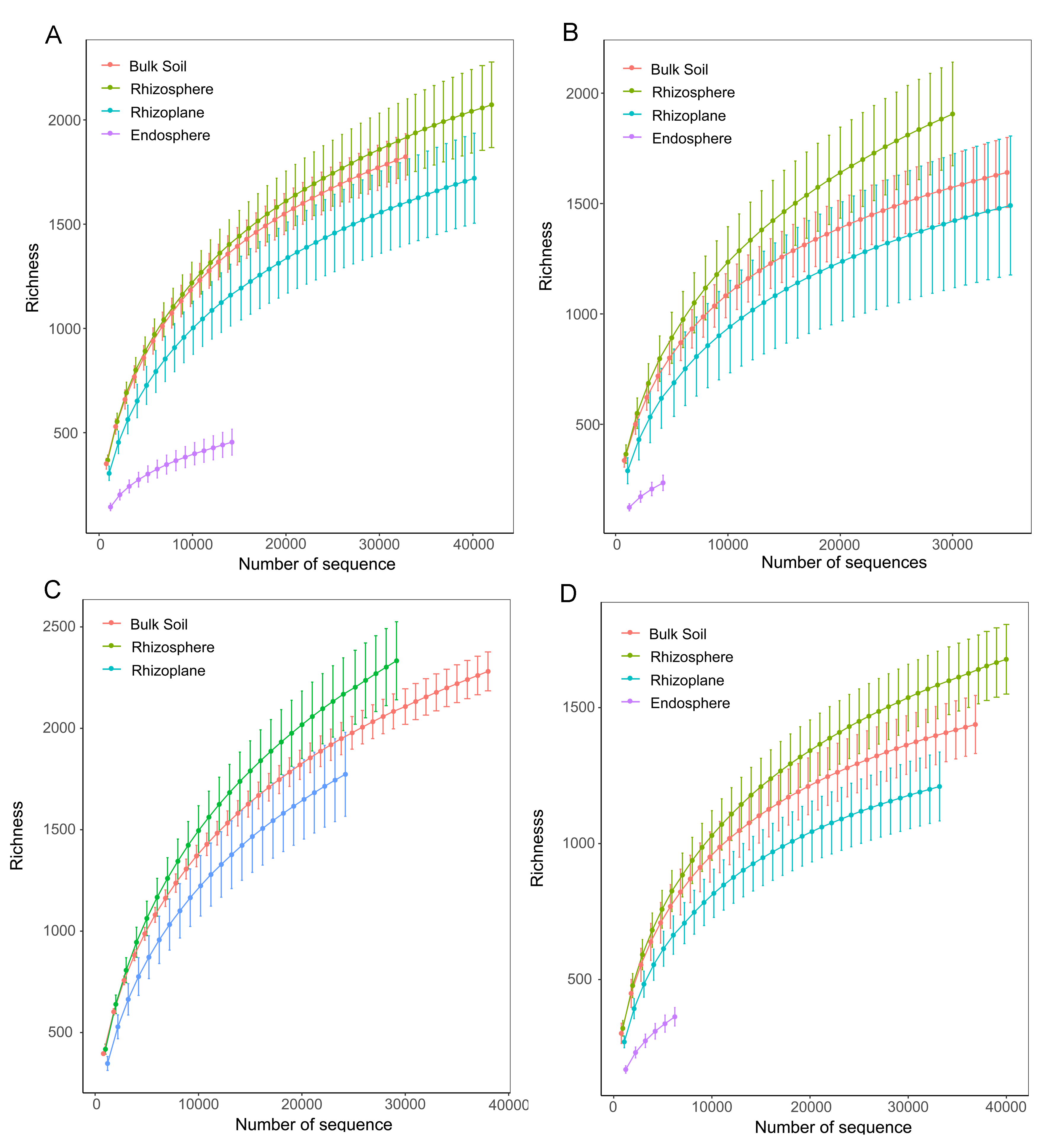

Supplement: Supplementary file 1 [file microorganisms-10-01644-s001.zip › Figure S1. Rarefaction curve of 16S rDNA showed that the number of OTUs increased with the number of sequences obtained in bulk soil, rhzizosphere, rhizoplanem and endosphere samples.tif]

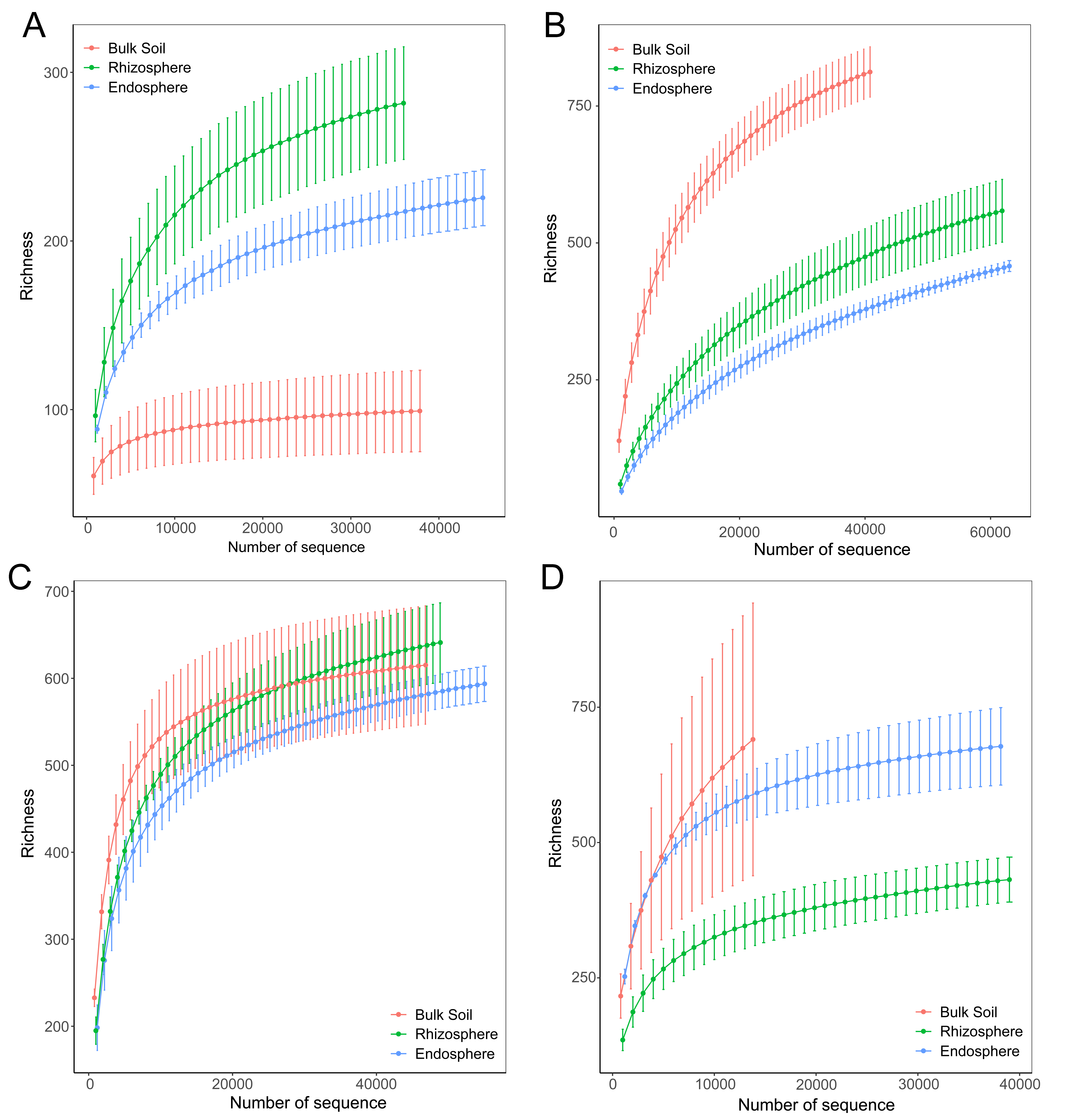

Supplement: Supplementary file 1 [file microorganisms-10-01644-s001.zip › Figure S2. Rarefaction curve of 18S rDNA showed that the number of OTUs increased with the number of sequences obtained in bulk soil, rhzizosphere, endosphere samples.tif]

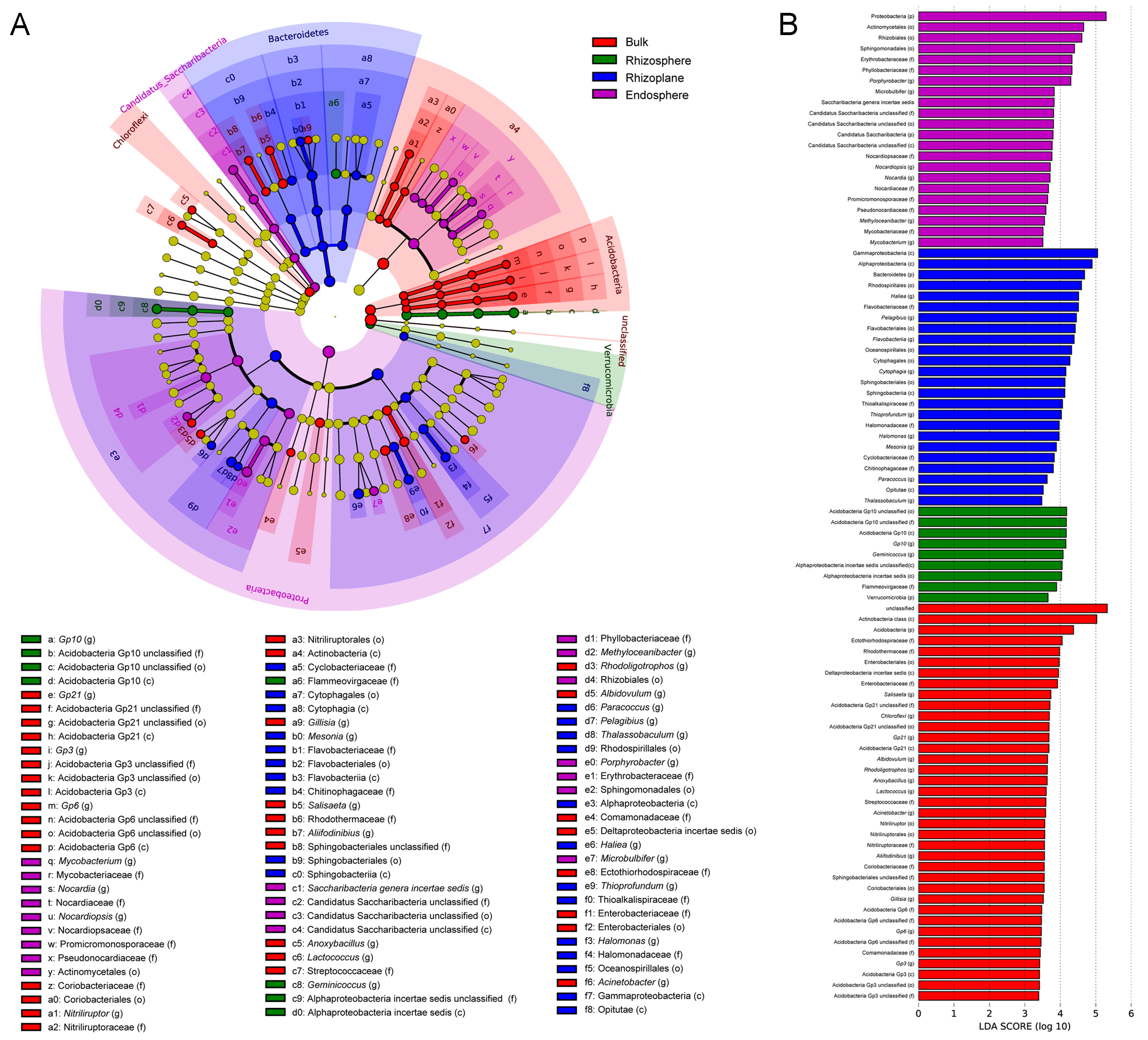

Supplement: Supplementary file 1 [file microorganisms-10-01644-s001.zip › Figure S7. LEfSe was used to identify rhizocompartment-specific (bulk soil, rhizosphere, rhizoplane, and endosphere) bacterial biomarkers in vegetative stage.tif]

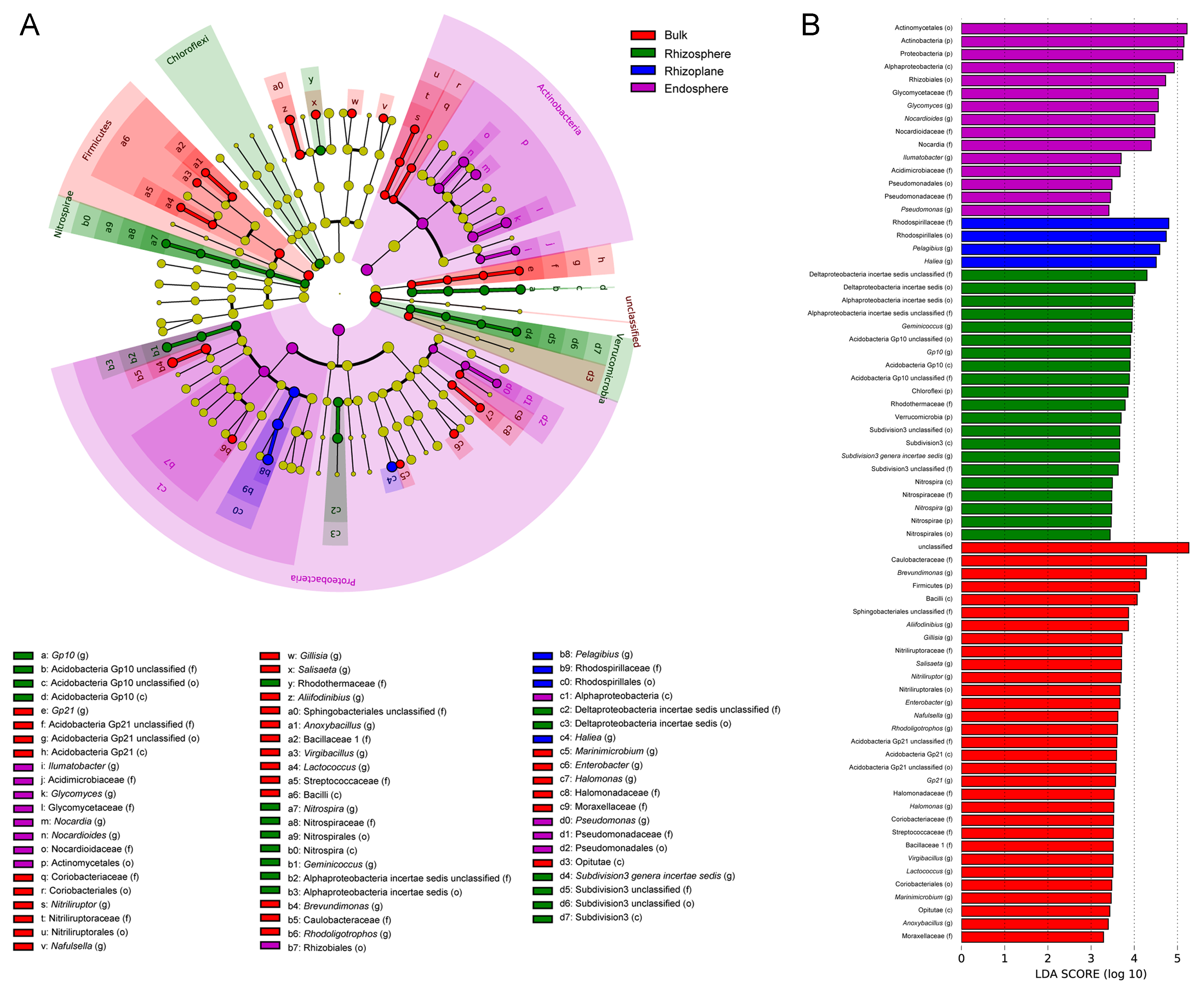

Supplement: Supplementary file 1 [file microorganisms-10-01644-s001.zip › Figure S8. LEfSe was used to identify rhizocompartment-specific (bulk soil, rhizosphere, rhizoplane, and endosphere) bacterial biomarkers in flowering stage.tif]

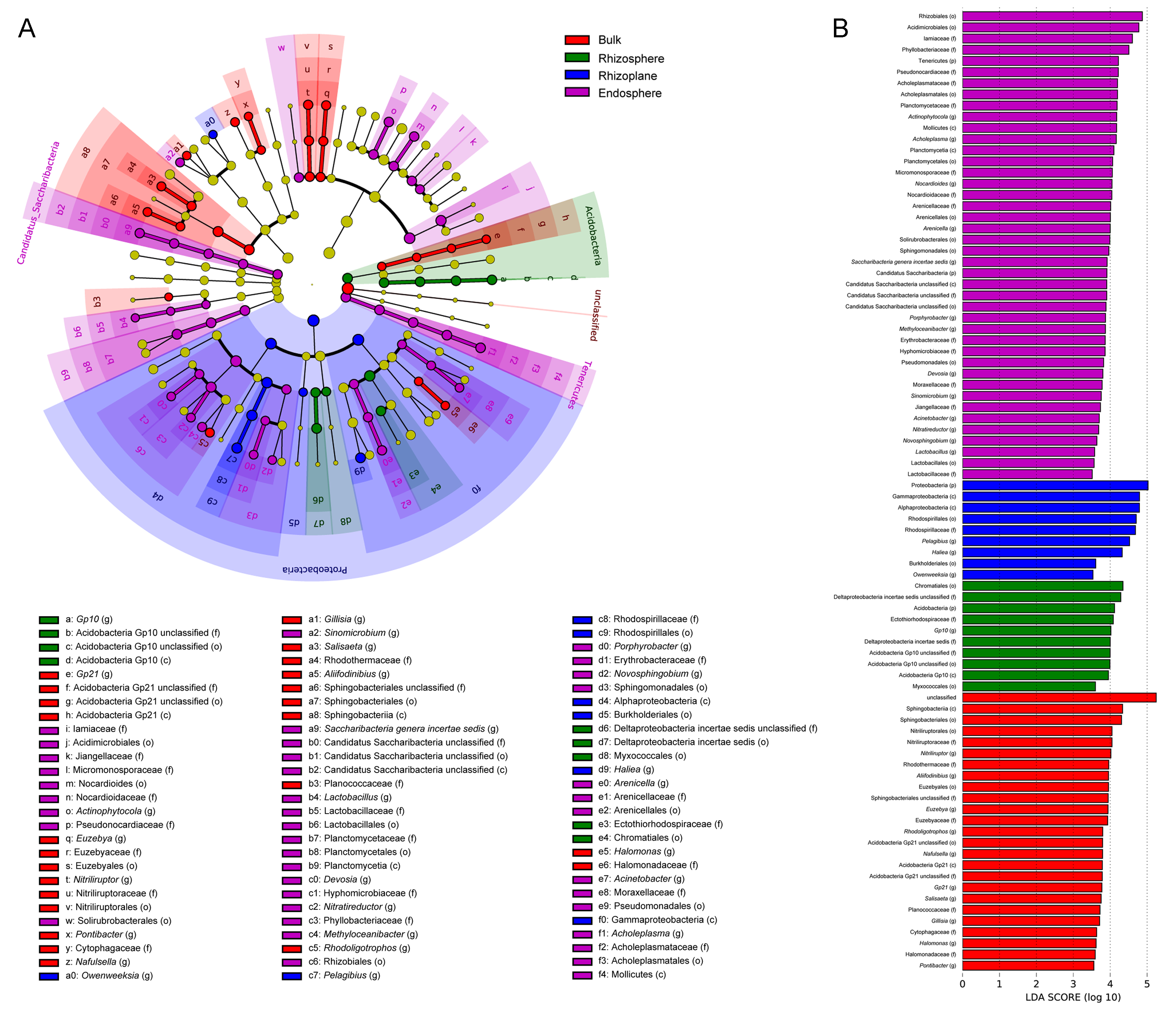

Supplement: Supplementary file 1 [file microorganisms-10-01644-s001.zip › Figure S9. LEfSe was used to identify rhizocompartment-specific (bulk soil, rhizosphere, rhizoplane, and endosphere) bacterial biomarkers in fruiting stage.tif]

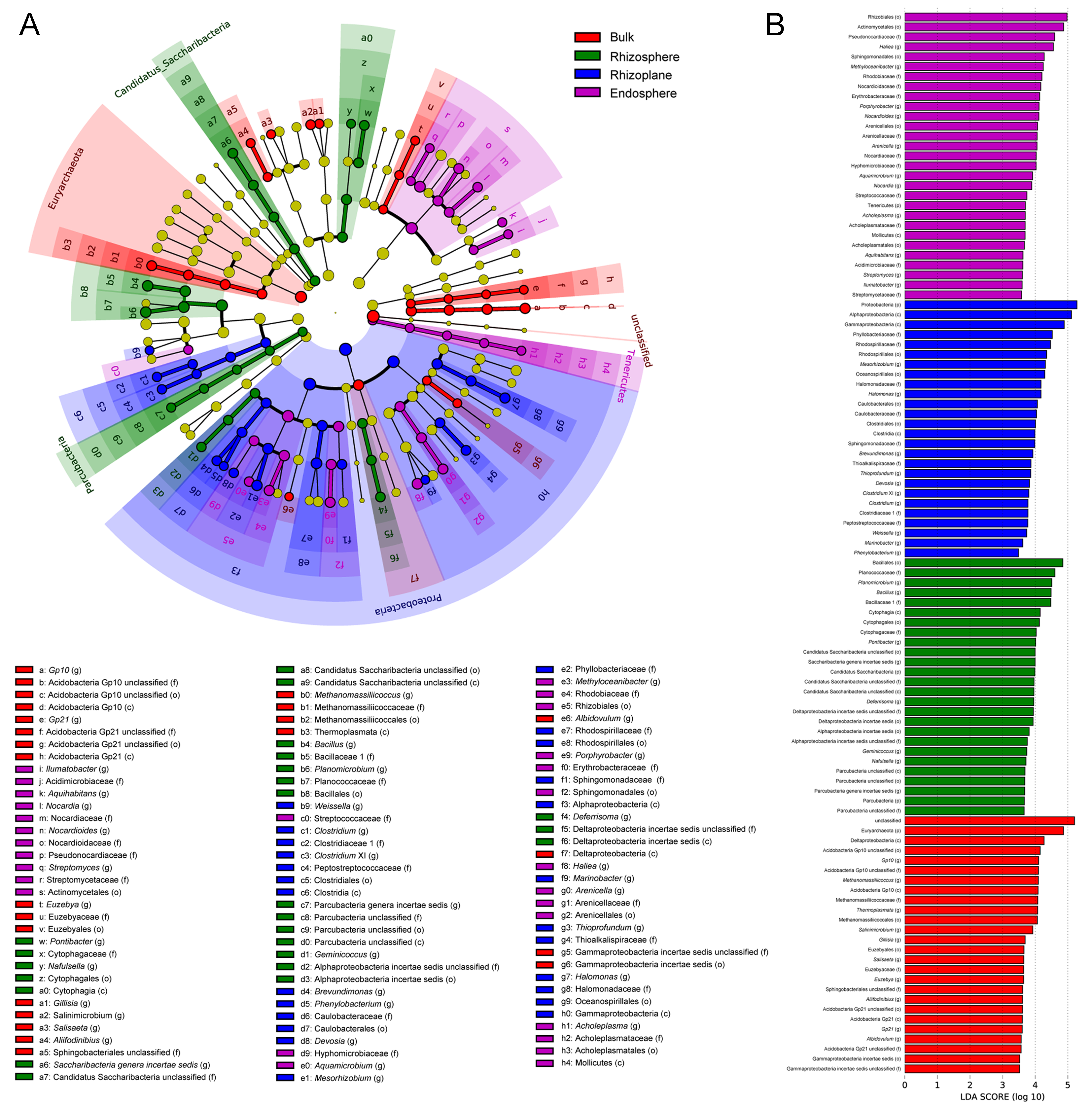

Supplement: Supplementary file 1 [file microorganisms-10-01644-s001.zip › Figure S10. LEfSe was used to identify rhizocompartment-specific (bulk soil, rhizosphere, rhizoplane, and endosphere) bacterial biomarkers in senescence stage.tif]

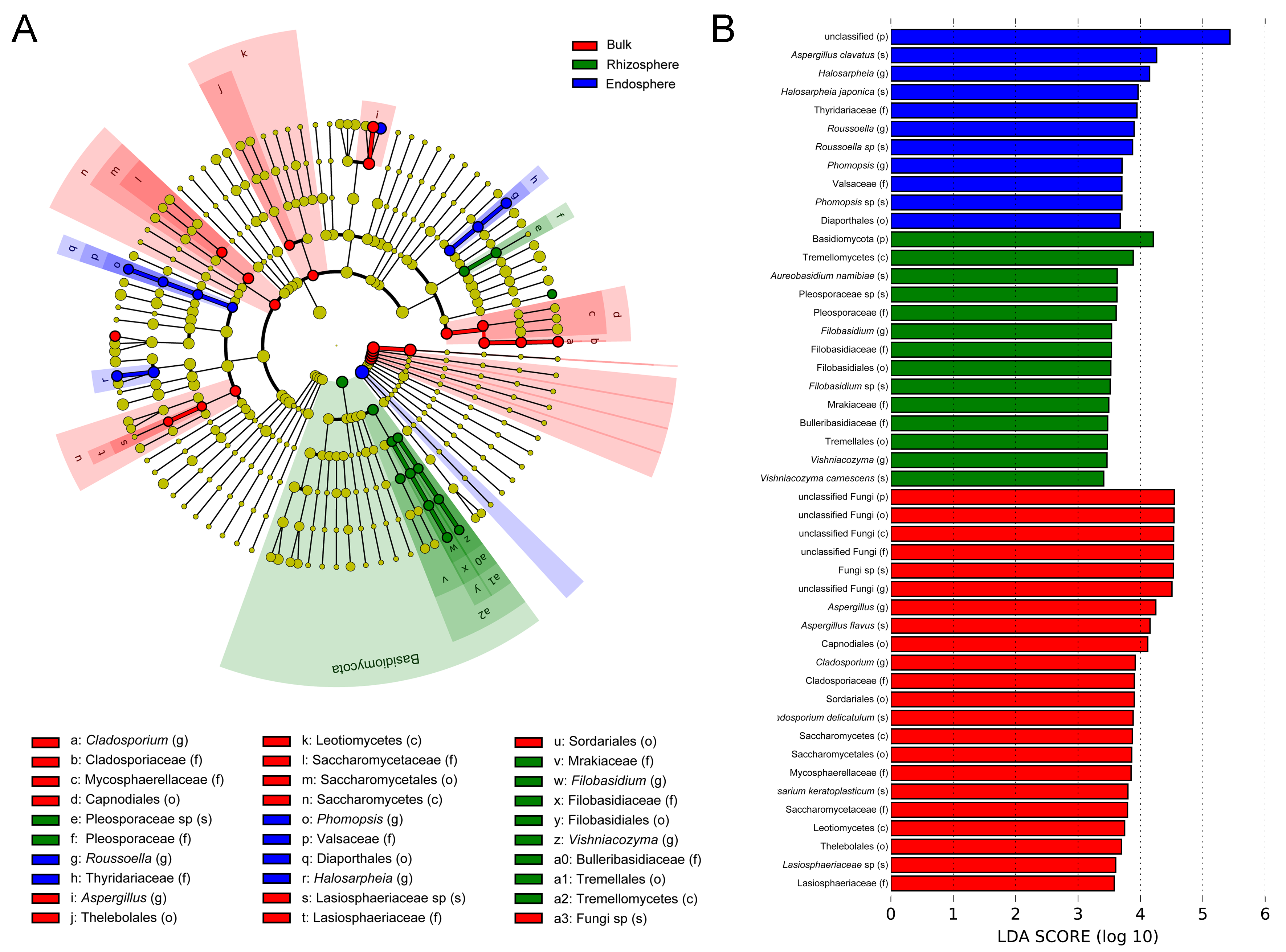

Supplement: Supplementary file 1 [file microorganisms-10-01644-s001.zip › Figure S11. LEfSe was used to identify rhizocompartment-specific (bulk soil, rhizosphere, and endosphere) fungal biomarkers in vegetative stage.tif]

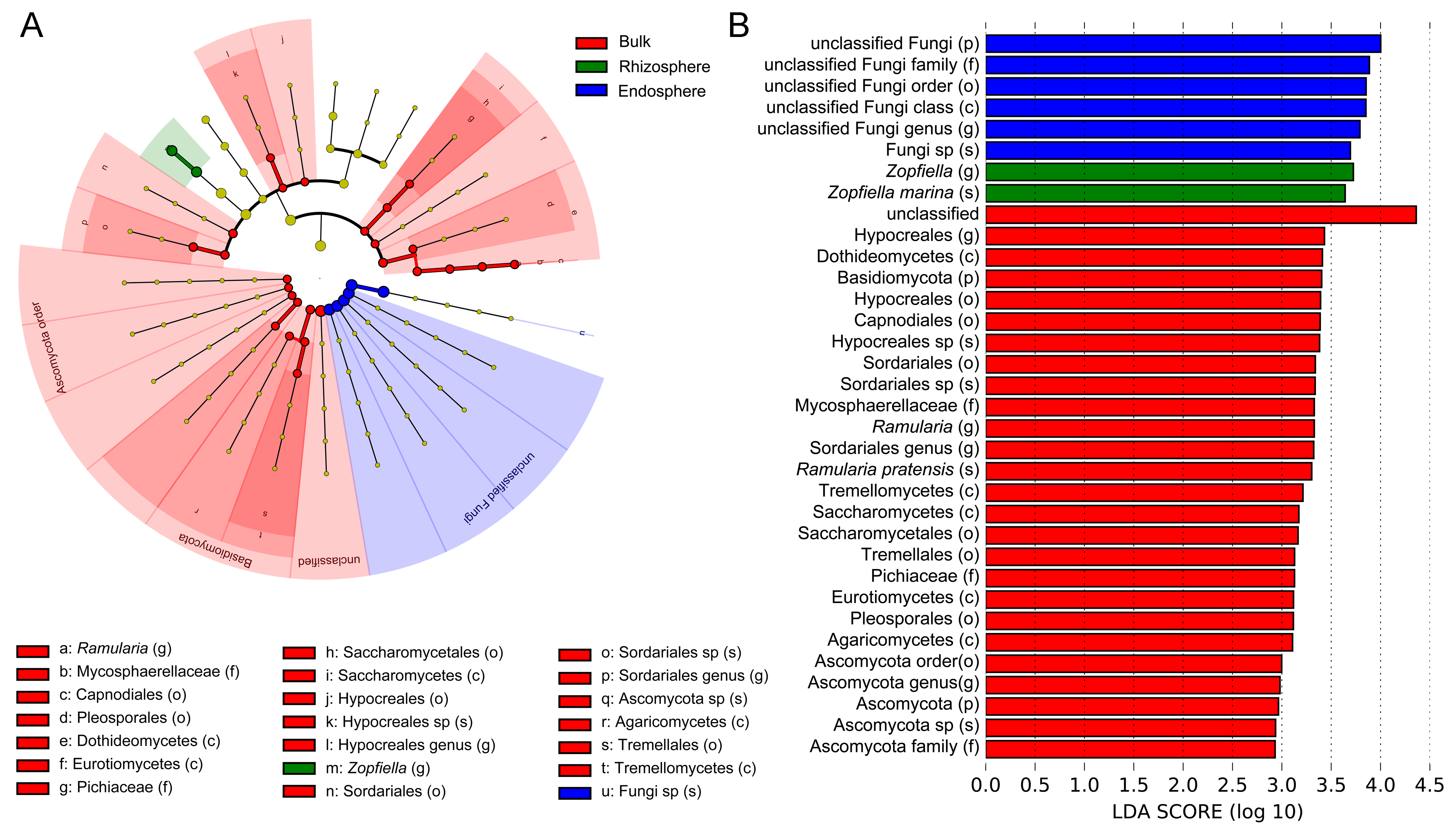

Supplement: Supplementary file 1 [file microorganisms-10-01644-s001.zip › Figure S12. LEfSe was used to identify rhizocompartment-specific (bulk soil, rhizosphere, and endosphere) fungal biomarkers in flowering stage.tif]

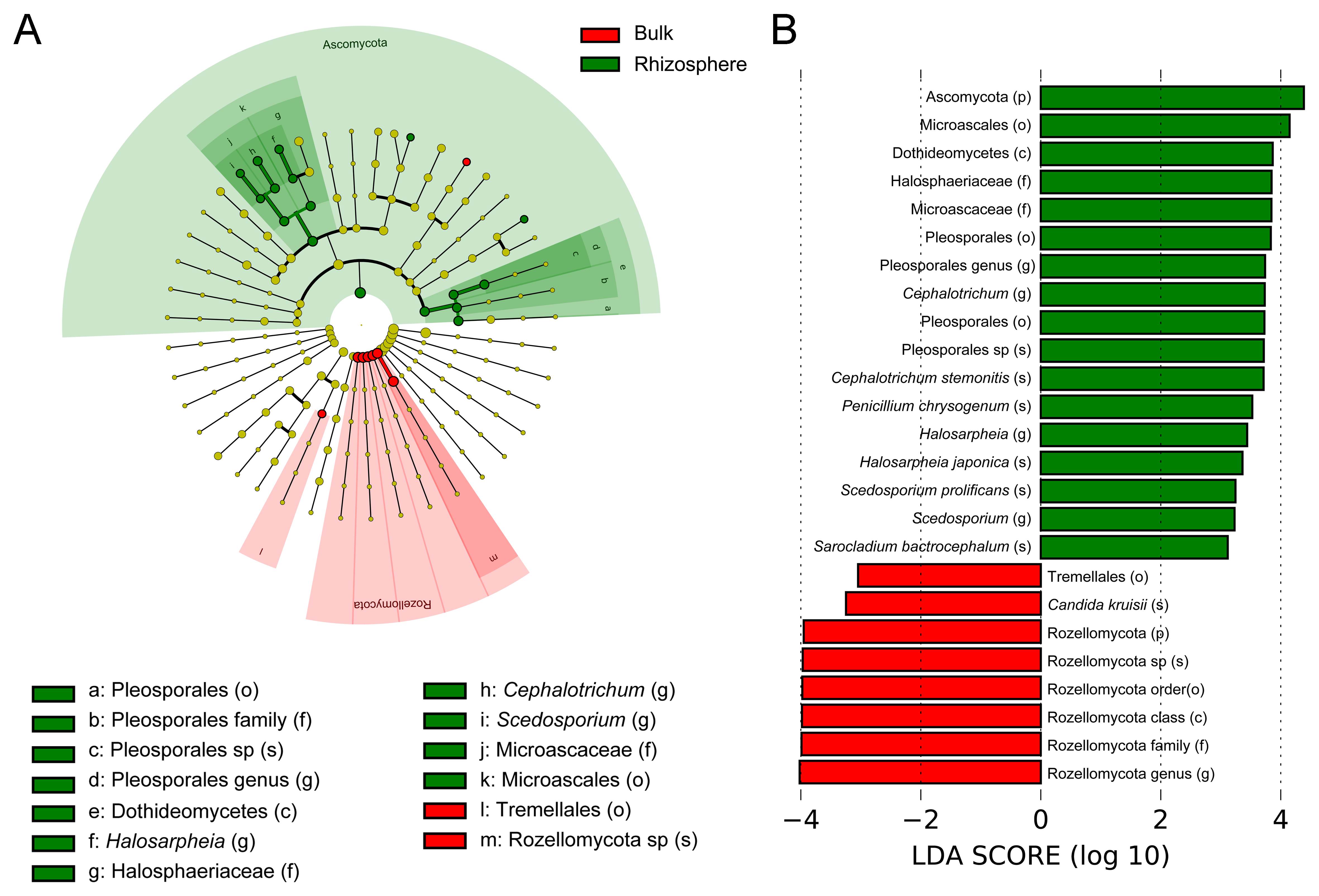

Supplement: Supplementary file 1 [file microorganisms-10-01644-s001.zip › Figure S13. LEfSe was used to identify rhizocompartment-specific (bulk soil, rhizosphere, and endosphere) fungal biomarkers in fruiting stage.tif]

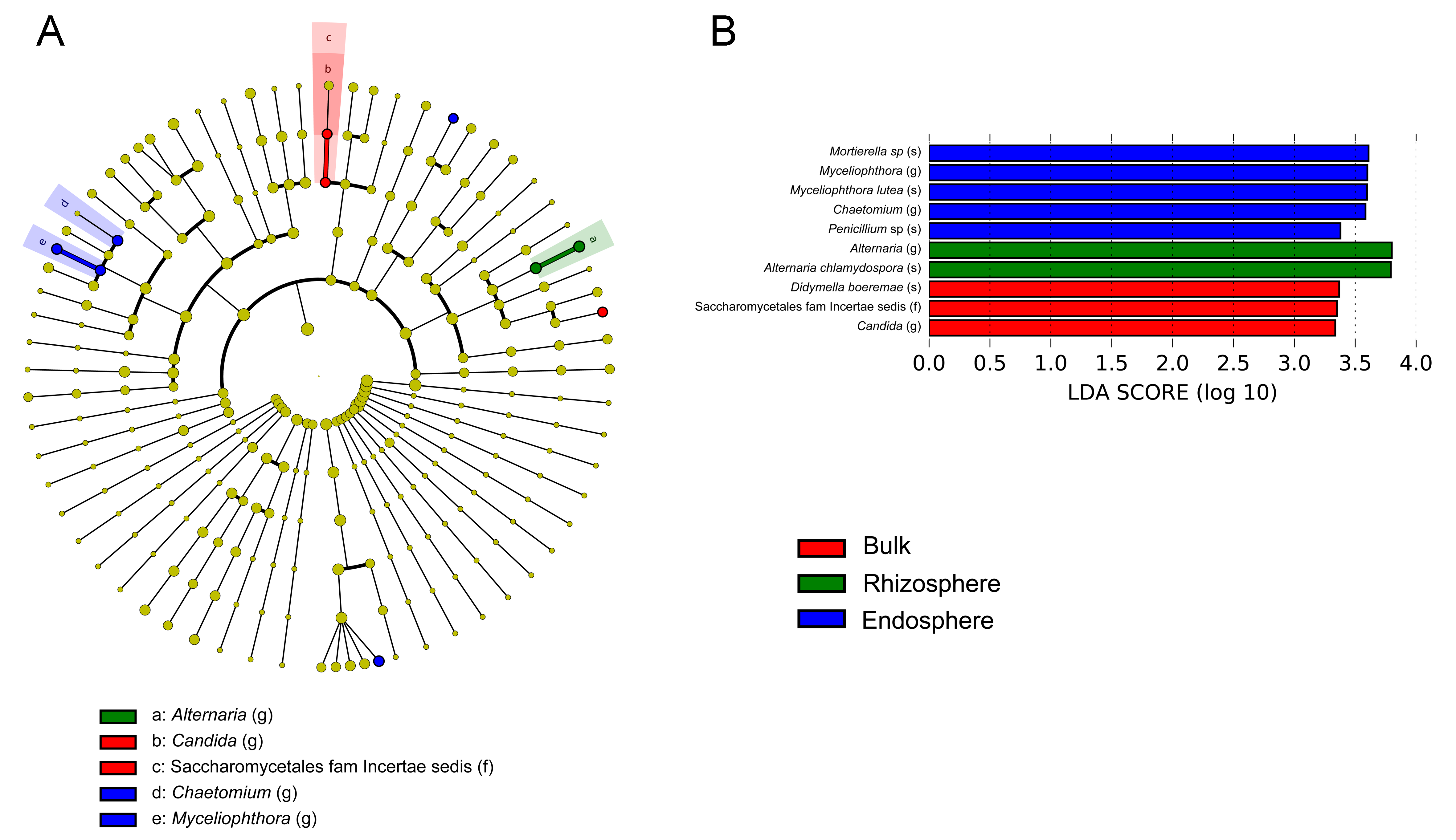

Supplement: Supplementary file 1 [file microorganisms-10-01644-s001.zip › Figure S14. LEfSe was used to identify rhizocompartment-specific (bulk soil, rhizosphere, and endosphere) fungal biomarkers in senescence stage.tif]

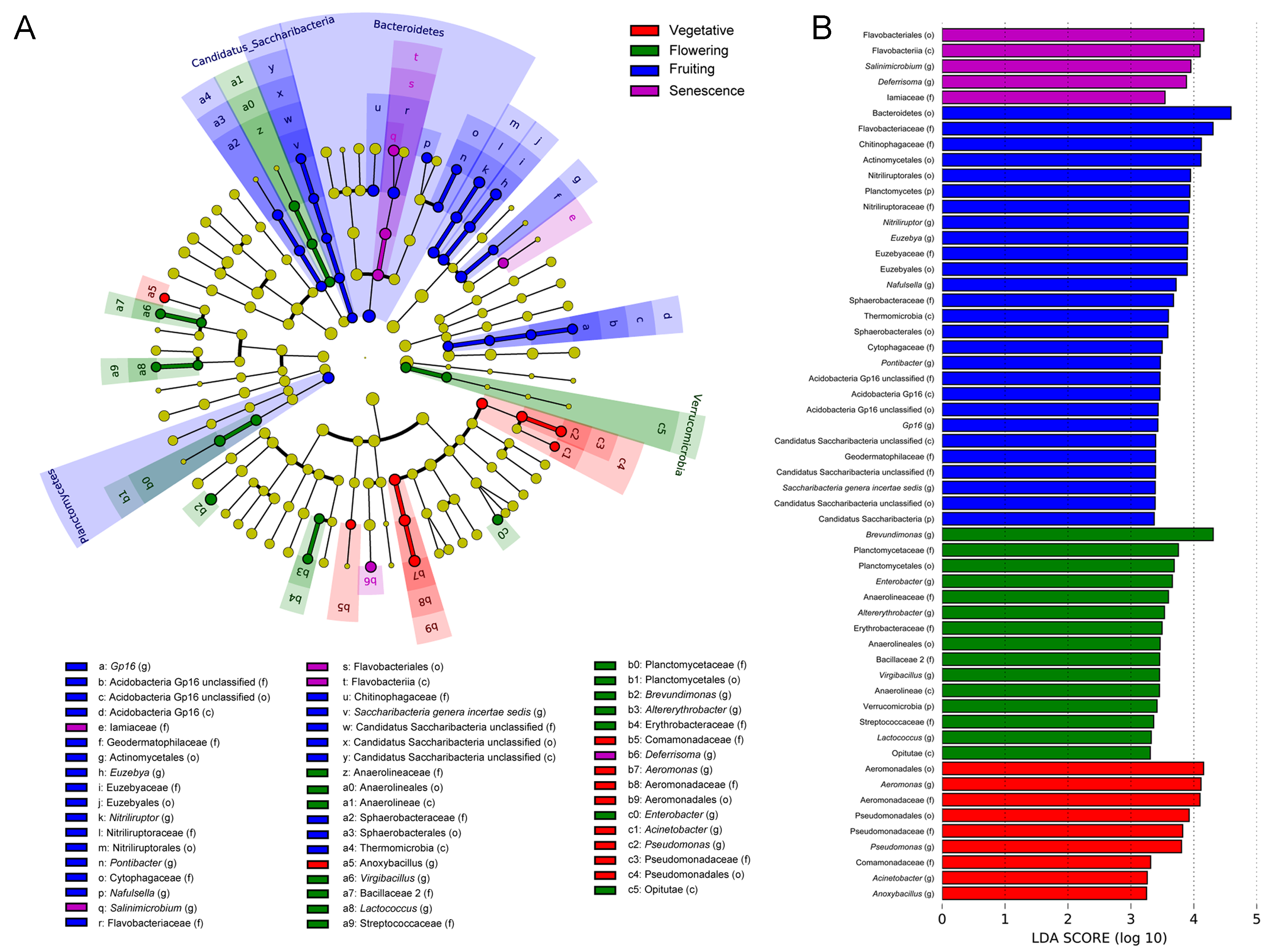

Supplement: Supplementary file 1 [file microorganisms-10-01644-s001.zip › Figure S15. LEfSe was used to detect growth stage-specific bacterial biomarkers in bulk soil communities.tif]

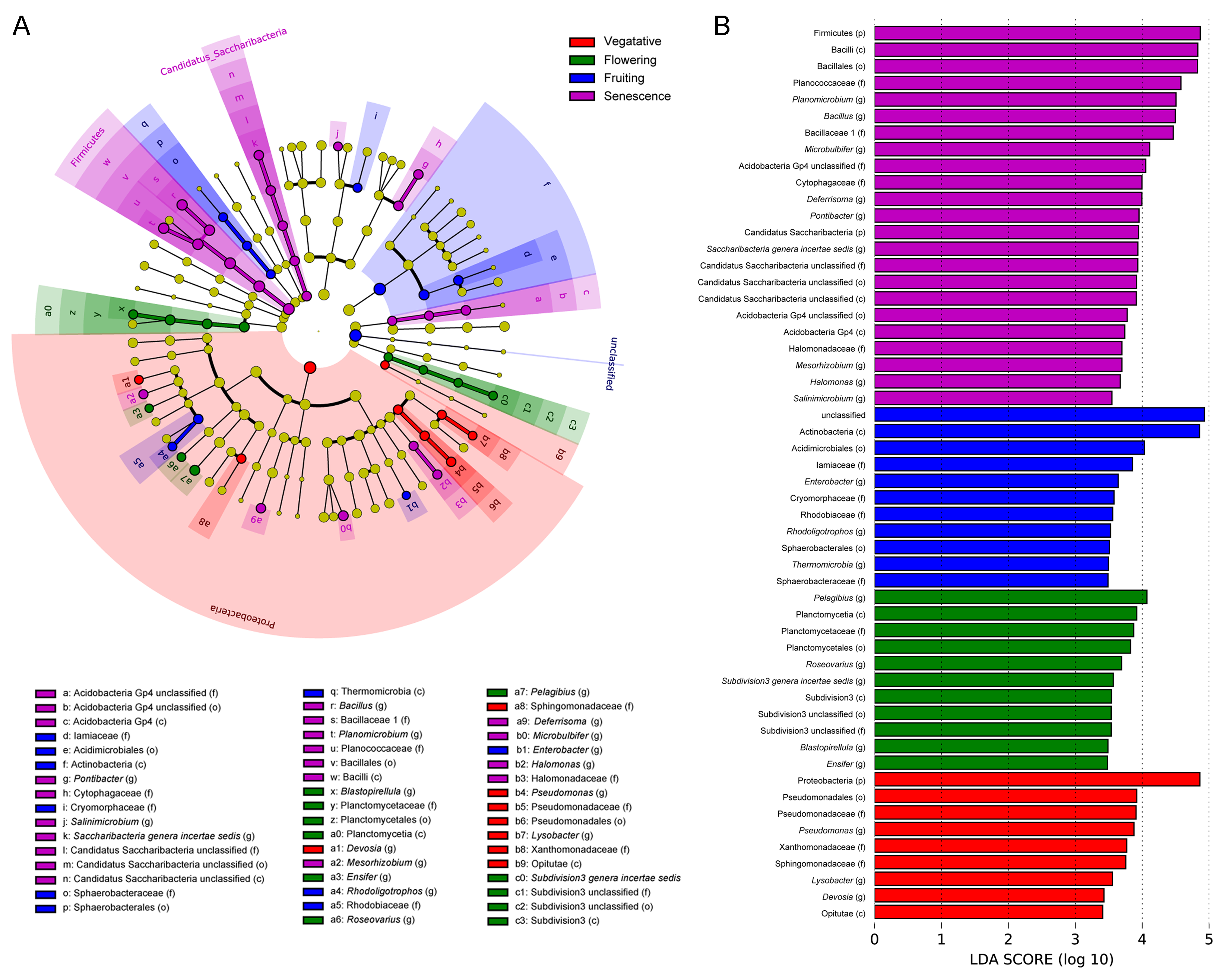

Supplement: Supplementary file 1 [file microorganisms-10-01644-s001.zip › Figure S16. LEfSe was used to detect growth stage-specific bacterial biomarkers in rhizospheric soil com-munities.tif]

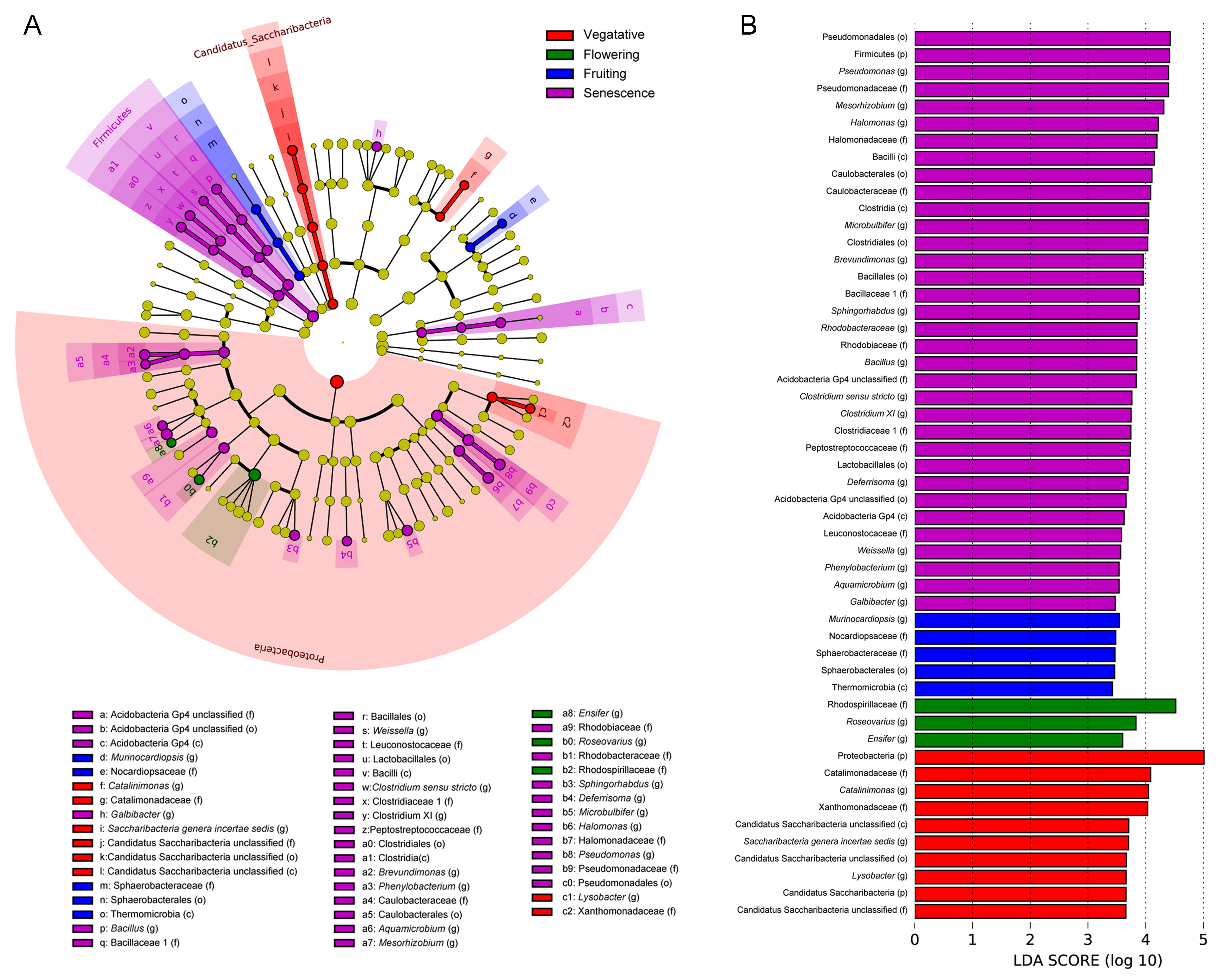

Supplement: Supplementary file 1 [file microorganisms-10-01644-s001.zip › Figure S17. LEfSe was used to detect growth stage-specific bacterial biomarkers in rhizoplane soil commu-nities.tif]

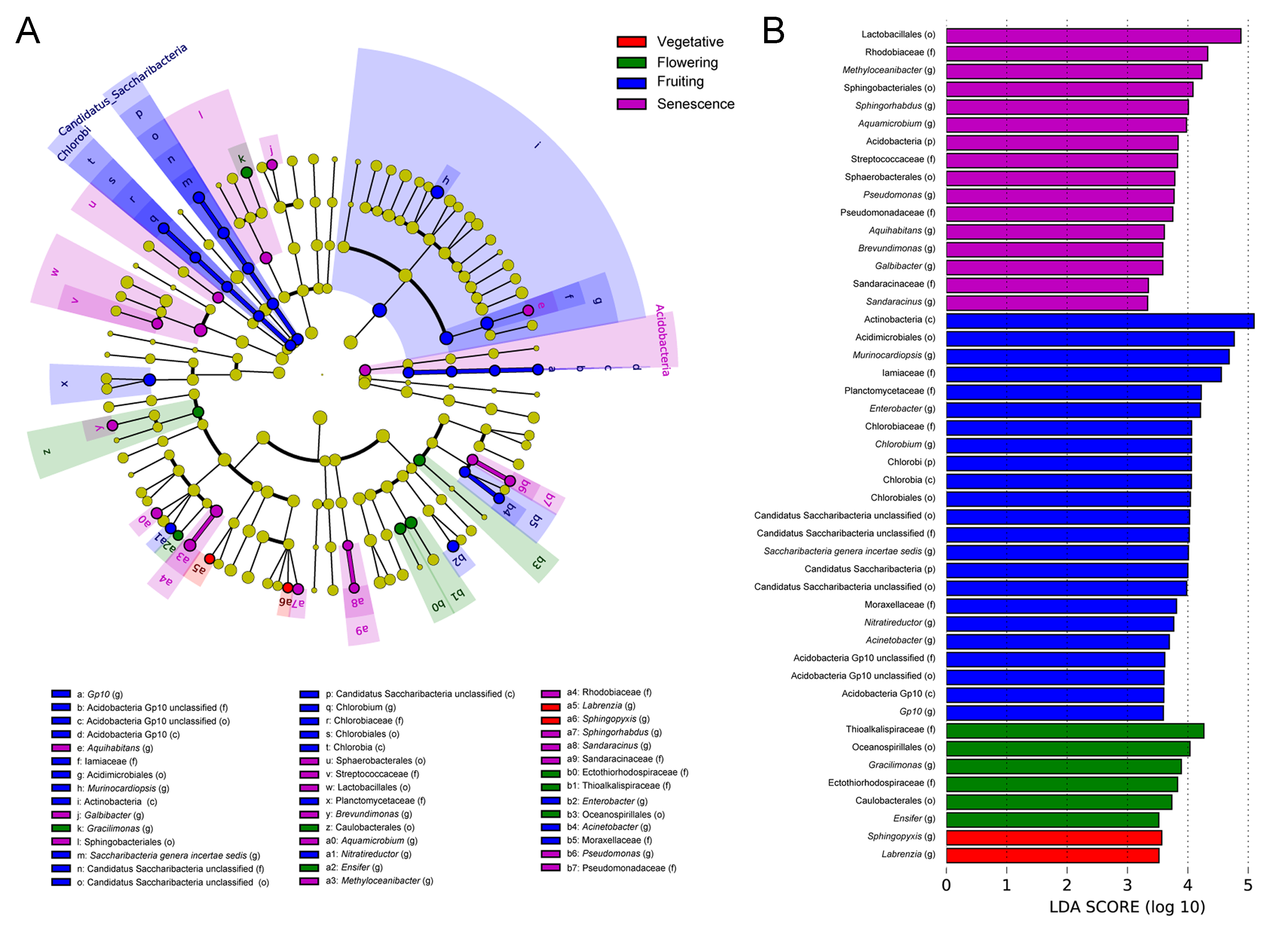

Supplement: Supplementary file 1 [file microorganisms-10-01644-s001.zip › Figure S18. LEfSe was used to detect growth stage-specific bacterial biomarkers in endospheric communities.tif]

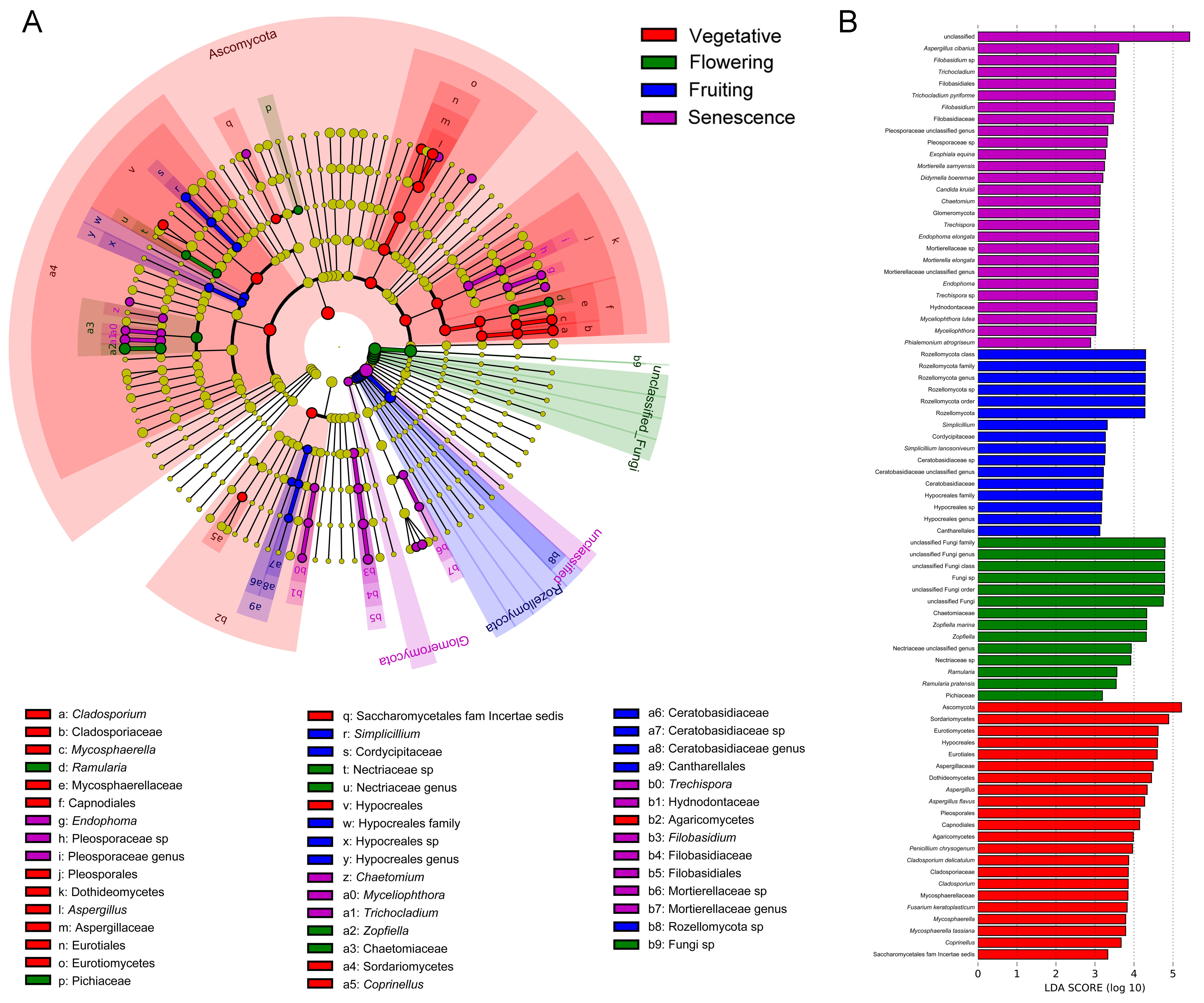

Supplement: Supplementary file 1 [file microorganisms-10-01644-s001.zip › Figure S19. LEfSe was used to detect growth stage-specific fungal biomarkers in bulk soil communities.tif]
